# Engineering Adaptive Immunity in 3D: A Patient‐Specific Lymphoid Model Using Stromal Networks and Peripheral Blood Mononuclear Cells

**DOI:** 10.1002/advs.202513245

**Published:** 2025-12-16

**Authors:** Mei ElGindi, Shaza Karaman, Jeremy Teo

**Affiliations:** ^1^ Laboratory for Immuno Bioengineering Research and Applications Division of Engineering New York University Abu Dhabi Abu Dhabi POBox 129188 UAE; ^2^ Department of Mechanical and Biomedical Engineering New York University Brooklyn NY 11201 USA

**Keywords:** immunoengineering, personalized therapy, stromal‐immune crosstalk, tertiary lymphoid organs (TLOs), tissue engineering

## Abstract

Tertiary lymphoid organs (TLOs) are non‐encapsulated immune structures that emerge in response to chronic inflammation, orchestrating local adaptive immune responses. However, recapitulating their complexity in vitro remains challenging due to the difficulty in generating  physiologically relevant stromal‐immune interactions. Here, a 3D lymphoid tissue model is presented, engineered using human adipose‐derived stem cells (ADSCs) differentiated into fibroblastic reticular cell (FRC)‐like populations within collagen matrices. Differentiation is induced using TNF‐α and LT‐α, with or without IL‐4, generating two stromal phenotypes: FRC_P1_ and FRC_P2_. These subsets exhibit matrix remodeling, distinct transcriptional signatures, and surface markers consistent with lymph node‐resident T cell reticular and follicular dendritic cell subsets. Upon co‐culture with peripheral blood mononuclear cells (PBMCs) and SARS‐CoV‐2 S1‐primed mature dendritic cells, the model supports antigen‐specific B cell activation and cytokine environments indicative of Th1 or Th2 polarization. FRC_P1_ favors B cell support and IgM secretion, whereas FRC_P2_ promotes dendritic cell activation and Th1‐type chemokine expression. This platform demonstrates the functional diversification of stem cell‐derived FRC‐like subsets and their role in orchestrating immune microenvironments. It enables investigation of lymphoid tissue remodeling, stromal‐immune crosstalk, and antibody generation using total PBMCs, providing a scalable, customizable system for personalized vaccine screening, autoimmune modeling, and therapeutic development.

## Graphical abstract

This study presents a 3D lymphoid tissue model engineered from adipose‐derived stem cells differentiated into fibroblastic reticular cell–like networks and co‐cultured with immune cells. The engineered system successfully generates antigen‐specific antibodies and cytokine responses, providing a platform for studying adaptive immunity, vaccine efficacy, and personalized immunotherapy in vitro.

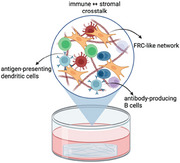

## 1 Introduction

Tertiary lymphoid organs (TLOs) are inducible lymphoid structures that emerge in non‐lymphoid tissues in response to chronic inflammation or infection. Although their structural organization is less ordered compared to secondary lymphoid organs like lymph nodes (LNs), they are still able to generate immune responses against antigens within specialized microenvironments created by a critical network of specialized stromal cells, such as fibroblastic reticular cells (FRCs).^[^ ^1^ ^]^ FRCs are not a homogeneous population of cells, and include follicular dendritic cells (FDCs) and T cell zone reticular cells (TRCs) that differ in their expression of cytokines, chemokines, and function.^[^ ^2^ ^]^ While TLOs lack a capsule, they can mature from small aggregates of T and B cells into clusters composed of T cell compartments, germinal centers, and B cell follicles, depending on the type of infection and the tissue in which they are located.^[^ ^1^, ^3^ ^]^ Beyond providing structural support, the stromal network regulates immune cell migration, facilitates antigen presentation and cytokine signaling, and promotes lymphocyte survival and differentiation.^[^ ^4^ ^]^

Tissue injury leads to local inflammatory responses and the release of pro‐inflammatory cytokines. These signals originate from infiltrating immune cells and resident fibroblasts, which then stimulate nearby stromal cells, including fibroblasts, myofibroblasts, and endothelial cells.^[^ ^5^ ^]^ This stimulation prompts local fibroblasts to proliferate and produce chemokines associated with lymphoid stromal cells, specifically CXCL13, CXCL12, and CCL19.^[^ ^1^, ^5^ ^]^ CXCL13 plays a key role in the recruitment and organization of B cells and some T cell subsets by binding to their CXCR5 receptors, while CXCL12 interacts with CCR7 to aid in lymphocyte trafficking and homing of memory CD8 T cells.^[^ ^6^ ^]^ CCL19 similarly attracts B and T cells, as well as dendritic cells (DCs), by engaging with their CCR7 receptors, directing these cells to lymphoid sites.^[^ ^1^ ^]^ The release of these chemokines by specialized fibroblasts enhances immune cell recruitment and promotes further production of chemokines and cytokines. In this inflammatory milieu, members of the tumor necrosis factor (TNF) superfamily, such as TNF‐α and lymphotoxin‐α (LTα), are expressed by activated immune cells and contribute to the induction and organization of lymphoid‐like structures. Although full activation of the lymphotoxin β receptor (LTβR) typically requires a heterotrimeric LTα_1_β_2_ complex provided by lymphoid tissue inducer (LTi) cells, studies have shown that LTα alone can still promote aspects of FRC differentiation and immune structuring, even without full LTβR engagement.^[^ ^7^, ^8^ ^]^ This process also leads to the formation of high endothelial venules (HEVs) and the organization of distinct B and T cell zones, which are indicators of mature TLO formation.^[^ ^9^, ^10^, ^11^ ^]^ Within B cell regions, FDCs secrete CXCL13, attracting T helper (Th) cells, which aid in the establishment of germinal centers, further promoting B cell proliferation and differentiation into antibody‐secreting plasma cells.^[^ ^12^ ^]^ The maintenance of these TLO structures relies heavily on sustained signals from DCs, as shown by experimental depletion of these cells in mouse models that resulted in the disintegration of TLOs.^[^ ^13^ ^]^

Despite the importance of FRCs and their critical role in supporting TLO immune responses, there are limited studies that develop TLO models that incorporate these cells in a physiologically relevant way. The use of traditional 2D in vitro models in these projects fails to capture the intricate 3D architecture of these structures, which is fundamental to the dynamic interactions within the stromal cell network. Higher complexity 3D models, which include the use of hydrogels and incorporate FRCs, provide a more physiologically relevant environment for studying immune responses. For instance, Morrison et al. developed a 3D LN model that incorporates FRCs and DCs, highlighting the critical role that FRCs play in supporting DC survival, proliferation, and localization within LNs.^[^ ^14^ ^]^ In addition, Kim et al. created a 3D hydrogel model of lymphoid organs by co‐culturing T cells and human bone marrow stromal cells, resulting in the formation of stromal networks with T cell attachment similar to in vivo T cell zones.^[^ ^15^ ^]^

While these models represent significant advancements, they still face limitations in fully capturing the complexity of the immune system. Many existing lymphoid models incorporate only specific cell types or focus on isolated aspects of immune function, such as T cell activation or B cell proliferation, without fully replicating the interaction between these processes. For example, Goyal et al. used primary T and B cells on a chip to form lymphoid follicles and study the antibody response to an influenza vaccine.^[^ ^16^ ^]^ Giese et al. went a step further and included mature dendritic cells (mDCs), along with T cells and B cells, to form a self‐assembling lymphoid tissue, however they still lacked the inclusion of stromal cells in their model.^[^ ^17^ ^]^ Recently, Zhong et al. generated germinal center organoids containing FDCs and B cells to specifically study B cell responses to antigen.^[^ ^18^ ^]^ To address these limitations, more comprehensive models that incorporate both the stromal cell network and diverse immune cell populations, as found in total peripheral blood mononuclear cells (PBMCs), are needed to capture the full spectrum of immune activities.

By engineering a 3D stromal network that more closely mimics the in vivo LN environment, we aim to create a robust platform for studying antibody production and immune function of PBMCs within lymphoid tissues, contributing to the development of immunotherapies and vaccines. Additionally, utilizing a stem cell‐derived stromal network rather than commercially available immortalized FRC lines addresses the scarcity of primary human FRC cells and allows flexibility in creating personalized TLO models tailored to specific therapeutic needs. Our study seeks to validate this model by demonstrating its ability to facilitate immune responses that support antibody production and enable the investigation of diverse immunological phenomena in a controlled, reproducible environment. Studies have shown that constructing these in vitro LN systems can enable the recall of specific memory B cell subsets that produce neutralizing antibodies against the COVID 19 virus.^[^ ^19^ ^]^ However, most existing models fail to integrate both the structural stromal network and total PBMCs, which are critical for supporting complex immune processes like specific antibody production against novel antigens.

Our tissue engineered fibroblastic reticular network, developed from adipose‐derived stem cells (ADSCs) embedded within 3D collagen hydrogels, demonstrated the presence of FRC‐like cells through surface marker expression. Further confirmation of these lymphoid‐associated stromal cells was subsequently obtained from transcriptome analysis, where immune and matrix remodeling processes were found to be enriched. The tissue engineering process revealed macroscopic remodeling of the collagen hydrogels, characterized by the contractile actions of these fibroblastic networks, an important feature, especially in lymph nodes, where the organ must possess mechanical strength to contain the expansion of immune cells during infections. Our tissue engineered fibroblastic tissues were shown to be heterogeneous and analyses of specific soluble cellular cues and microscopy revealed elements that actively direct immune cells. These networks further enhance immune responses, supporting antigen‐specific antibody production from immune cells spiked with SARS‐CoV‐2 proteins. This system lays a foundation for advanced research into lymphoid organs and immunology, with the potential to enhance therapeutic development.

## 2 Experimental Section

### 2.1 Cell Culture and FRC Differentiation

Human adipocyte‐derived stem cells (ADSCs), purchased from Thermofisher and ATCC, were maintained in MesenPRO RS medium (Thermo Fisher Scientific Inc., Dreieich, Germany) under standard cell culture conditions (37 °C, 95% humidity, and 5% CO2). For 3D cell culture experiments, collagen matrices were prepared by mixing type I collagen from rat tail (Advanced BioMatrix, Inc. San Diego, CA, USA), 0.1% acetic acid (Sigma–Aldrich, St. Louis, MO, USA), and 500 mm phosphate buffer (Sigma–Aldrich, St. Louis, MO, USA) at a concentration of 2 mg mL^−1^, following previously published protocols.^[^ ^20^ ^]^ To enable covalent binding of the collagen matrix via lysine side chains, the collagen solution was transferred onto a glutaraldehyde‐coated coverslip (13 mm in diameter; VWR, Darmstadt, Germany).^[^ ^21^ ^]^ Fibrillogenesis of the collagen matrix was achieved by placing the coverslips at 37 °C, 5% CO_2_, and 95% humidity. The 3D collagen matrices were then washed three times with phosphate buffer saline (PBS; Thermo Fisher Scientific Inc, Leicestershire, UK) and placed into 24‐well plates (Thermo Fisher Scientific Inc, Dreieich, Germany).

For FRC differentiation, the collagen matrices were then seeded with 1 x 10^5^ ADSC cells well^−1^ in a 1:1 mixture of MesenPRO RS and X‐VIVO 15 medium (Thermo Fisher Scientific Inc., Dreieich, Germany, and Lonza, Basel, Switzerland), and incubated overnight to allow for infiltration of the cells into the collagen matrices. The following day, the medium was removed, and the cells were cultured in standard conditions with X‐VIVO 15 medium supplemented with either 50 ng mL^−1^ IL‐4, 10 ng mL^−1^ TNF‐α, and 50 ng mL^−1^ LT‐α (for FRC_P1_) or 20 ng mL^−1^ TNF‐α, and 100 ng mL^−1^ LT‐α (for FRC_P2_). All activating cytokines were purchased from Biolegend, USA. A control group was maintained in X‐VIVO 15 medium without any supplements. The cultures were maintained for 10 days, with the medium changed on day 5.

To ensure stemness of ADSCs, each donor batch was differentiated, at the same passage as used in the experiments, into adipocytes by adding StemPro Adipocyte Differentiation Basal Medium (Gibco, Invitrogen, Thermo Fisher Scientific Inc., Dreieich, Germany) for 10 days. Medium was replaced every three days.

### 2.2 mDC Activation and Co‐Culture of FRCs with PBMCs and mDCs

For mature dendritic cell (mDC) differentiation, monocytes were separated from total PBMCs (purchased from AllCells, LLC) by allowing them to adhere to cell culture flasks in RPMI‐1640 cell culture medium supplemented with 10% fetal bovine serum (FBS), 1% HEPES, 1% sodium pyruvate, 0.01% beta‐mercaptoethanol, and 1% penicillin/streptomycin. Cell culture medium and supplements were purchased from Gibco, Invitrogen, Thermo Fisher Scientific Inc., Dreieich, Germany. Non‐adherent cells were then removed, and monocytes were washed once with PBS. RPMI‐1640 medium supplemented with 400 ng mL^−1^ IL‐4 and 100 ng mL^−1^ GM‐CSF was then added to induce differentiation into immature dendritic cells (iDC) cells over 6 days, with 50% of the medium being refreshed on day 3. Following this, the medium was removed and replaced with fresh medium supplemented with 1 µg mL^−1^ SARS‐CoV‐2 S1 protein (cat# 797006) to induce maturation into mature dendritic cells (mDCs) over 2 days. All activating cytokines and protein were purchased from Biolegend, USA.

For the co‐culture experiments, following the differentiation of ADSCs into FRC‐like cells, fresh X‐VIVO 15 medium was added to the wells along with 7.5 × 10^5^ PBMCs and 1 × 10^4^ mDCs from the same donor. These cells were co‐cultured for 15 days at standard cell culture conditions, with fresh X‐VIVO 15 medium being supplemented every 5 days. After the 15‐day period, supernatant was collected for cytokine, chemokine, and antibody analysis, while the cells were used for further analysis, such as flow cytometry or imaging.

### 2.3 Quantitative Analysis of Surface Markers and UMAP processing

Collagen matrices were digested by incubation with 2 mg mL^−1^ collagenase (Gibco, Thermo Fisher Scientific Inc., Dreieich, Germany) in PBS for 10 min at standard cell culture conditions. To quantify the expression of surface markers on FRCs, cells were stained with antibodies against CD21, ICAM1, PDPN, Thy1, CD44, LTBR, and VCAM1 for 30 min on ice in PBS.

For co‐culture cell surface marker analysis, samples were stained with two different antibody panels. For the FRC panel, antibodies against CD35, Thy1, CD157, CD105, RANKL, CD44, VCAM1, PDPN, ICAM1, and CD21 were used. For the PBMC panel, antibodies against CD3, CD8, CD4, CD69, PD1, CD14, CD11b, CD11c, CD68, CD86, HLADR, CD19, CD20, BAFFR, CD27, CD138, CD30, and CD38 were used. For B cell receptor specificity, samples were stained with CD14, CD19, CD3, CD4, and Streptavidin FITC (Cat#405202, concentration of 0.5 µg mL^−1^). All antibodies were purchased from Biolegend, USA. Viability stain Zombie Aqua was purchased from ThermoFisher (Cat# L34957) and used at a dilution of 1:2000 (Thermo Fisher Scientific, Inc., Dreieich, Germany). Details regarding clone, conjugated fluorochrome, dilution, isotype, and catalog number of the antibodies are provided in Table S1 (Supporting Information). Samples were stained in 50% PBS and 50% Brilliant Stain Buffer (Biolegend, USA). Afterward, stained cells were analyzed using the Cytek Aurora flow cytometer (4L 16V‐14B‐10YG‐8R) that utilizes an unmixing (compensation) program from the SpectroFlo software. Experiments were performed in at least 6 replicates, and analysis was performed using FlowJo software (Becton, Dickinson and Company, NJ, USA).

### 2.4 Imaging

Following ADSC differentiation and co‐culture with PBMCs and mDCs, samples were fixed with 4% paraformaldehyde (Biolegend, USA) for 10 min and then washed three times with PBS. Samples were then stained with Hoechst 33342 (Cat# H3570; 1:10000; Invitrogen, Carlsbad, CA, USA) and Flash Phalloidin Red 594 (Cat# 424203;1:1000; BioLegend, USA) in PBS overnight at 4 °C, followed by three PBS washes. Cell imaging was performed using either a BioTek Lionheart FX Automated Microscope (Agilent, USA) with a 4x objective or an epifluorescence microscope (DMi8 S; Leica, Germany) equipped with 10× and 40× long‐working‐distance objectives (NA 0.4; Leica, Germany). For 3D visualization, z‐stacks were acquired and reconstructed using the Leica LAS X software 3D viewer, and videos were generated along the z‐axis to visualize cluster formation.

To further assess ICAM‐1 and PDPN expression and PBMC clustering across samples, an additional immunofluorescence staining step was performed. The fixed samples were washed twice with PBS, quenched in Tris‐HCl (pH 8.0) for 15 min at room temperature (RT), and washed two times with PBS. Non‐specific binding sites were blocked with 2% bovine serum albumin (BSA) in PBS for 30 min at RT. Nuclei were stained with Hoechst 33342 (Cat# H3570; 1:10000; Invitrogen, Carlsbad, CA, USA) for 10 min at RT, followed by two PBS washes. Antibodies were diluted in PBS and incubated overnight at 4 °C. The following antibody mixtures were used: CD45–APC (Cat#: 555485; 1:50; BD Biosciences, USA), PDPN– CoraLite594 (Cat#: CL594‐67432; 1:10; Proteintech Group, USA), and ICAM‐1–Alexa Fluor 488 (Cat#: 353130; 1:200; BioLegend, USA). After incubation, samples were washed twice with PBS. Confocal imaging was performed using an Olympus IX83 confocal microscope equipped with a 20× objective under identical acquisition parameters for all samples.

To visualize and track the shrinking of the collagen gels and the formation of clusters, brightfield images of the full wells were captured using the BioTek Lionheart FX Automated microscope at a ×4 objective without staining every 24 h for the full 25‐day experiment. The area of the collagen gel in these images was then measured in square pixels (px2) using ImageJ software.^[^ ^22^ ^]^

### 2.5 RNA Isolation and Purification

RNA was extracted using TRIzol (Thermo Fisher Scientific, Inc., Dreieich, Germany), followed by chloroform extraction (Sigma–Aldrich, Schnelldorf, Germany) using the manufacturer's protocol. Afterward, purification using the RNeasy mini kit (Qiagen, Hilden, Germany) was performed according to the manufacturer's protocol. The obtained RNA concentration and purity (the ratio of absorbance at 260 nm and 280 nm) were quantified using a Nanodrop (Thermo Fisher Scientific, Inc., Dreieich, Germany) and confirmed by the Qi RNA kit with Qubit 4 fluorometer (Thermo Fisher Scientific, Inc., Dreieich, Germany) prior to performing RNA sequencing.

### 2.6 RNA Sequencing and Analysis

RNA‐sequencing was performed with Dante Lab genomics (New York, USA), which was given the RNA samples and provided the raw counts data. RNA‐Seq data were then merged using the NASQAR toolbox (publicly accessible at http://nasqar.abudhabi.nyu.edu/; accessed on: 18th June 2024). Analysis was performed using iDEP 2.01 (http://bioinformatics.sdstate.edu/idep/ (last accessed on: 17th October 2024), publicly accessible by South Dakota State University) to determine the top enriched pathways using the GAGE method (FDR ≤ 0.2).

### 2.7 Cytokine and Chemokine Quantification Analysis

To study the cytokine and chemokine production by the co‐cultured samples, cell culture supernatant was collected at the endpoint of the experiment, and the secretion of IL‐4, GM‐CSF, IL‐1β, TNF‐α, IL‐2, IL‐7, IFN‐γ, CXCL10, CXCL9, CXCL11, CX3CL1, IL‐5, IL‐10, IL‐13, CCL19, CXCL12, and CXCL13 were analyzed using a custom‐made bead‐based ELISA from LEGENDPlex as well as other LEGENDPlex ELISA kits listed in **Table** **1** below (Biolegend, San Diego, CA, USA). Experiments were performed in at least 6 replicates.

**Table 1:** LEGENDplex cytokine and chemokine kit information. All purchased from Biolegend.

Product Name	Catalog Number
HU Th Cytokine Panel (12‐plex)	741027
HU Essential Immune Response Panel (13‐plex)	740929
HU Proinflam. Chemokine Panel 1 (13‐plex)	740984
HU Proinflam. Chemokine Panel 2 (12‐plex)	741157

### 2.8 Immunoglobulin Specificity and Quantification Analysis

To determine the specificity and quantity of IgM and IgG against SARS‐CoV‐2 S1 protein, the supernatant was collected at the endpoint of the experiment. In addition, after collagenase was used to digest the matrices, the samples were centrifuged, and the supernatant of this process was also collected and used for immunoglobulin analysis. LEGEND MAX SARS‐CoV‐2 Spike S1 Human IgM ELISA and LEGEND MAX SARS‐CoV‐2 Spike S1 Human IgG ELISA kits were used to determine the concentration of antigen‐specific antibody in the conditions (Cat# 448207 and 447807, respectively, Biolegend, San Diego, CA, USA). Experiments were performed in at least 6 replicates.

### 2.9 Data and Statistical Analysis

Statistical significance was determined using an unpaired one‐way ANOVA followed by the Kruskal‐Wallis post‐hoc test with Dunn's test for multiple comparisons using Prism 10 Version 10.2.3 (GraphPad Software Inc., San Diego, USA), with significance set to *p* ≤ 0.05. Unless otherwise stated, all experiments were performed in at least four replicates. Data were presented as mean ± standard deviation (SD).

## 3 Results and Discussion

### 3.1 Effects of Lymphotoxin‐α, Tumor Necrosis Factor‐α, and IL‐4 on Stem Cell‐Derived Stromal Differentiation

**Figure 1:**
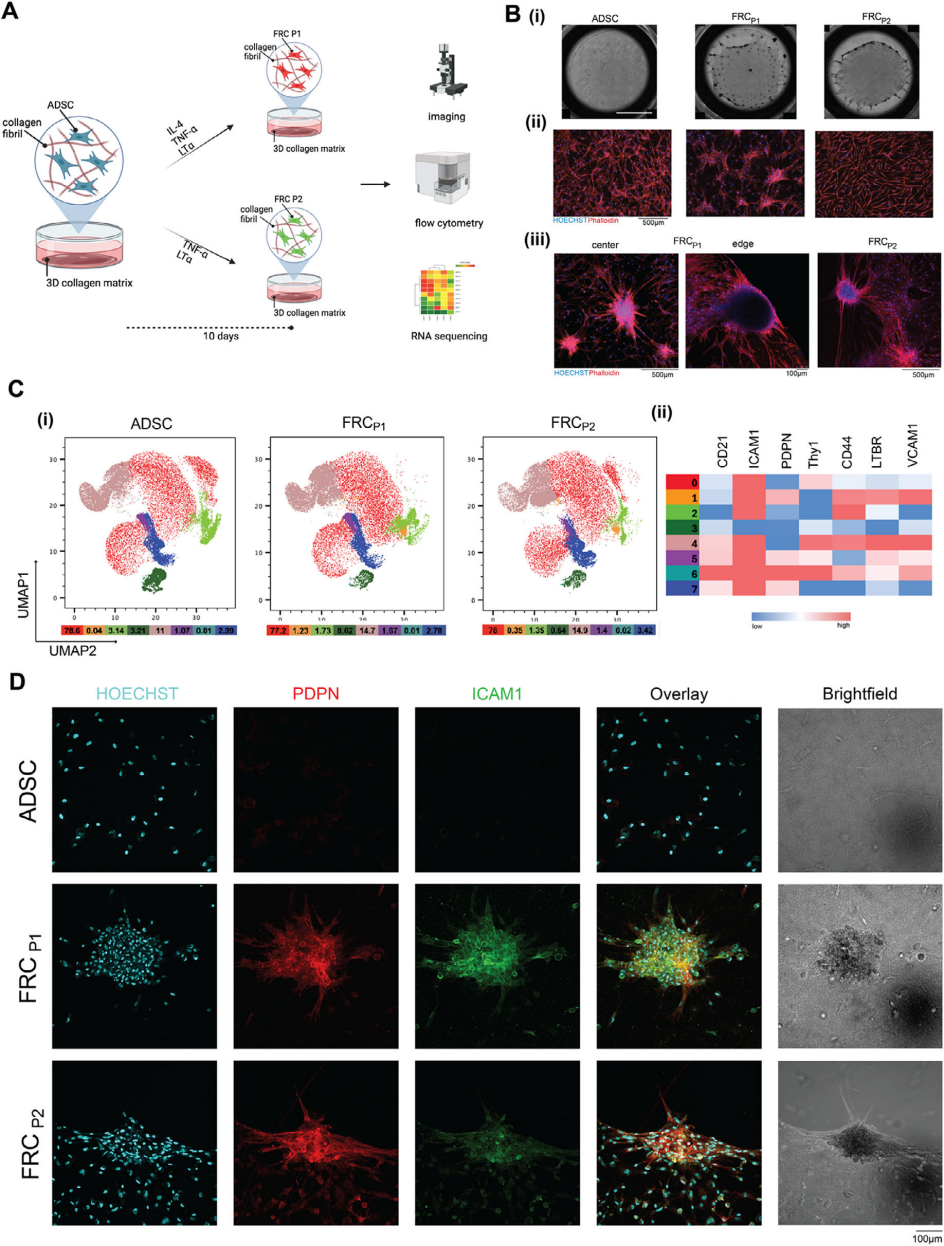
Differentiation of Adipose Derived Stem Cells (ADSC) into Fibroblastic Reticular‐like Cells (FRC). A) Schematic of approaches, P1 & P2, used to differentiate ADSCs into FRC‐like cells. The 10‐day differentiation was followed by analysis using imaging, flow cytometry, and RNA‐sequencing. Created in https://BioRender.com B) i) Representative images showing full well images of ADSCs, FRC_P1_, and FRC_P2_ cells after 10 days of culture within collagen matrices. Scale bar: 5 mm ii) Images taken at x4 on Lionheart microscope stained with Phalloidin 594 (red) and Hoeschst (blue) showing the differences in morphology of the three cell types. Scale bar: 500 µm iii) Images taken at x10 on LEICA STED stained with Phalloidin 594 (red) and Hoeschst (blue) of FRC_P1_ and FRC_P2_. Scale bar: 500 µm. A 3D rotational video of the FRC_P1_ edge appears as a video in the Supporting Information. C) i) Flow cytometry derived UMAP plot of populations in ADSCs, FRC_P1_, and FRC_P2_ cells after 10 days of differentiation. Each plot represents 16 concatenated samples from four distinct ADSC donors. Values below each UMAP represent the percentage of each subpopulation within the treatment condition. Colors correspond to the subpopulation in the heatmaps. ii) Heat map showing the expression levels of CD21, ICAM1, PDPN, Thy1, CD44, LTBR, and VCAM1 in each population. Values are normalized within each column relative to the specific marker. D) Representative images taken at 20x on Olympus IX83 with immunofluorescence staining of ADSCs, FRC_P1_, and FRC_P2_ for nuclear marker Hoechst (cyan), PDPN (red), and ICAM1 (green). Overlays demonstrate co‐localization of PDPN and ICAM1 within multicellular clusters.

There exists a growing body of literature that highlights that adipose‐associated lymphoid tissues, although less structurally organized relative to LNs, contain similar cellular components, including lymphoid stromal fibroblastic reticular cells (FRCs) that form the fibroblastic reticular network.^[^ ^23^, ^24^ ^]^ These FRCs likely differentiate from the mesenchymal cells within the stromal vascular fraction.^[^ ^25^ ^]^ Based on these insights, we sought to derive FRC‐like cells from adipose‐derived stem cells (ADSCs), using a differentiation protocol that combines LT‐α and TNF‐α, with an additional variant using IL‐4. Importantly, our protocol does not engage the lymphotoxin‐β receptor (LTβR), as LT‐α in its homotrimeric form (LTα_3_) does not activate LTβR.^[^ ^26^ ^]^ Rather, we aimed to examine whether stimulation with TNF‐α and LT‐α, with or without IL‐4, is sufficient to promote an FRC‐like phenotype in human ADSCs. IL‐4 was included in our differentiation protocol based on its reported ability to enhance fibroblast proliferation, extracellular matrix remodeling, and the expression of adhesion molecules and chemokines that support lymphocyte interaction, which are features that are functionally relevant to FRC biology.^[^ ^27^, ^28^ ^]^ We termed the resulting populations FRC_P1_ (TNF‐α + LT‐α + IL‐4) and FRC_P2_ (TNF‐α + LT‐α), while undifferentiated ADSCs served as our control group. To verify that the ADSCs retained their stemness characteristics and ability to differentiate into specific cell types, we tested each donor batch of our cells prior to experimental use by inducing differentiation into adipocytes. Representative images in Figure S1 (Supporting Information) demonstrate successful adipogenic differentiation, confirming that the cells maintained their pluripotency and were suitable to be used for further experiments. In addition, we tested the various components in our differentiation protocol individually as well as in alternative combinations (TNF‐α + IL‐4, and LT‐α+ IL‐4). Data from flow cytometry, seen in Figure S2 (Supporting Information) shows that indeed, our FRC_P1_ and FRC_P2_ protocols result in the cells having the highest levels of ICAM1, PDPN, and VCAM1, which are critical markers of FRC‐like cells.^[^ ^29^ ^]^ Several studies have attempted to develop in vitro FRC models using signaling molecules to trigger appropriate pathways in different stromal cell types.^[^ ^30^, ^31^, ^32^ ^]^ However, no study has comprehensively compared the effectiveness of different activation approaches using multi‐resolution analyses, including transcriptomics, protein expression, secretion profiles, and functional assays. In this study, the differentiation of ADSC into FRC‐like cells was conducted using two combinations of different cytokines at varying concentrations, within 3D collagen matrices, which better recapitulate the fibrous and mechanically dynamic environment of lymphoid tissues compared to traditional 2D surfaces or in biomaterials originating from hydrocarbons.^[^ ^33^ ^]^ Following a 10‐day differentiation period, the cells were analyzed using microscopy, flow cytometry, and RNA sequencing (**Figure** **1**A).

Microscopy revealed noticeable tissue‐level morphological differences between FRC_P1_ and FRC_P2_ cells in comparison to ADSCs. In particular, differentiated cells were observed to cluster and exhibit what appears to be remodeling and reorganization of the lab‐based matrices (Figure 1B i). Distinctively, FRC_P1_ cells formed clusters that are interspersed throughout the collagen matrices, whereas FRC_P2_ cells formed clusters that favor the edge of the gels, with dispersed individual cells exhibiting a more elongated shape relative to ADSCs (Figure 1B ii). Figure 1B iii presents magnified and fluorescently labeled depictions of the clusters that form post differentiation in FRC_P1_ and FRC_P2_. FRC_P1_ clusters appeared in the center and the edge of the matrix, and a 3D video of the edge image can be found in the Supporting Information.

Through UMAP analysis derived from flow cytometry measurement of cell surface markers, we obtained an overview of the distribution of cellular subpopulations within undifferentiated and differentiated cell populations of ADSCs (Figure 1C i). Supporting this, a heat map illustrates the relative expression levels of the various surface markers, while the respective subpopulation percentages can be seen under each figure (Figure 1C i,ii). Subpopulation 1 expressed high ICAM1, CD44, LTBR, and VCAM1, but low CD21, indicating an activated T‐zone reticular cell (TRC)‐like or FRC‐like cells.^[^ ^34^ ^]^ Subpopulation 2 displayed high ICAM1 and CD44 with low PDPN and VCAM1, similar to splenic stromal cells that recognize hyaluronan and help in the retention and function of plasma cells.^[^ ^35^ ^]^ Subpopulation 4 expressed high levels of all markers except PDPN, suggesting a non‐canonical follicular dendritic cell (FDC)‐like population that is highly activated or in a mature state, potentially involved in immune regulation or antigen capture.^[^ ^36^ ^]^ Subpopulation 6 showed high expression of CD21, ICAM1, PDPN, Thy1, CD44, and VCAM1, with moderate LTBR, aligning closely with follicular dendritic cells (FDCs) that are vital for supporting germinal center formation and facilitating the production of high‐affinity antibodies.^[^ ^34^, ^36^ ^]^ In contrast, subpopulation 7 expressed CD21, ICAM1, and PDPN but was low in other markers, consistent with a marginal reticular cell (MRC)‐like identity.^[^ ^36^ ^]^ Subpopulation 0 was defined primarily by ICAM1 expression and likely represents early stromal progenitors or residual ADSCs.^[^ ^36^ ^]^ Subpopulation 3, low across all markers, likely reflects non‐activated cells, while subpopulation 5 expressed all measured markers except CD44, suggesting a subtype of MRC‐like cells that are less migratory, explaining the low levels of CD44.^[^ ^37^ ^]^ To further validate the phenotypic differences between ADSCs, FRC_P1_, and FRC_P2_ cells, we performed immunofluorescence staining for nucleus (Hoechst), PDPN, and ICAM1. Representative images are shown in Figure 1D. In ADSC controls, expression of both PDPN and ICAM1 was negligible, consistent with their undifferentiated phenotype. In contrast, FRC_P1_ and FRC_P2_ displayed strong induction of both markers, with FRC_P2_ showing notably higher PDPN expression relative to FRCP1. ICAM1 was similarly upregulated in both differentiated populations compared to ADSCs. Overlay images demonstrate clear co‐localization of PDPN and ICAM1 within multicellular clusters, supporting the presence of FRC‐like phenotypes in both differentiated groups, with FRC_P2_ adopting a particularly PDPN‐enriched profile. Alone, surface marker analyses is insufficient to define the specific FRC subtypes, and therefore require further evaluation. Nonetheless, from a surface marker point of view, we are able to deduce that we can obtain FRC‐like cells from ADSCs using our differentiation protocol. Furthermore, these FRC‐like cells self‐assembled and organized into 3D spatial networks, formed unique cell‐clustered structures, and demonstrated the ability to be contractile and remodel the extracellular matrix (ECM), which are additional characteristics inherent in FRCs.^[^ ^38^, ^39^ ^]^

### 3.2 Transcriptome of Fibroblastic Reticular‐Like Cells Differentiated from Adipose‐Derived Stem Cells

**Figure 2:**
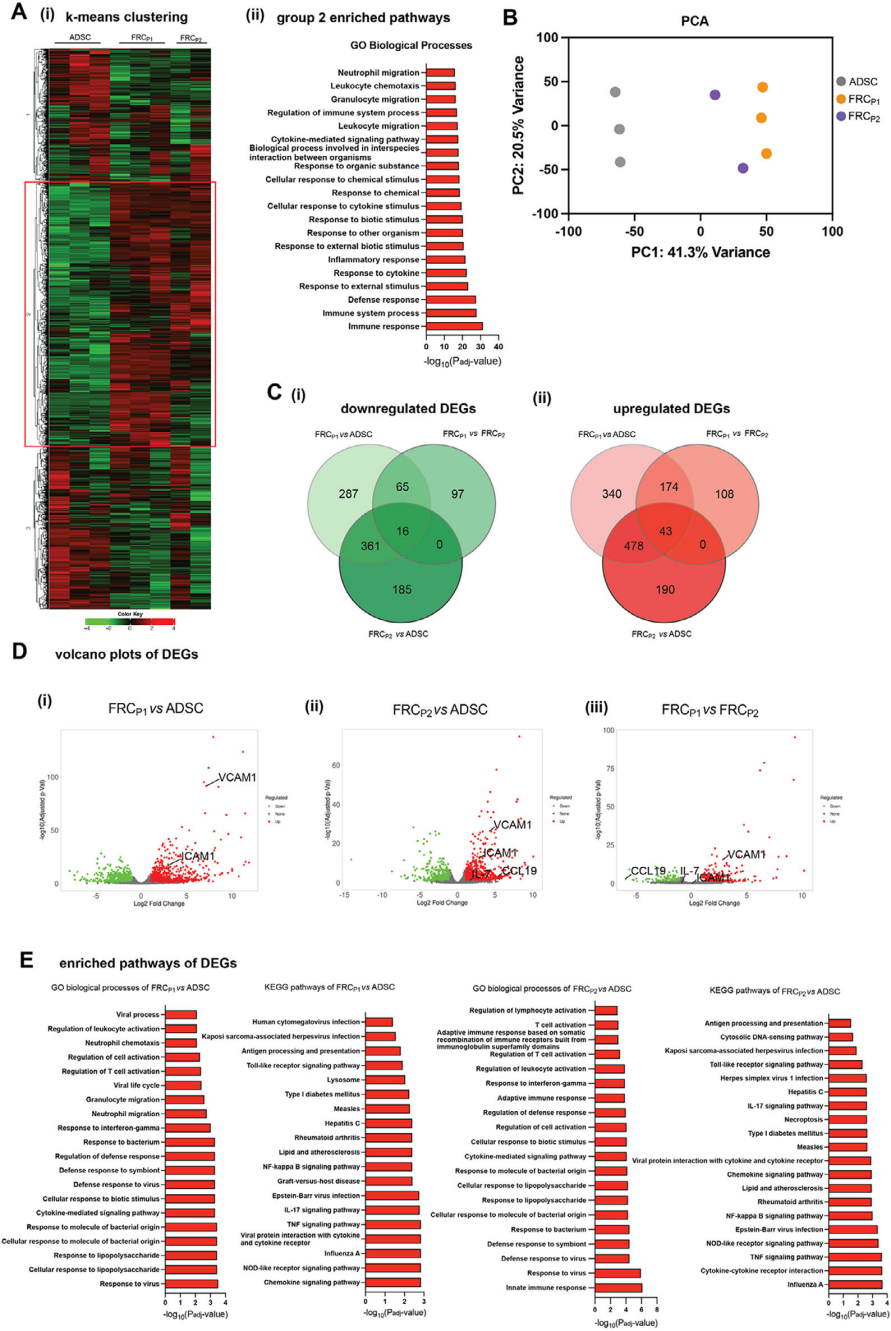
Transcriptomic Differences in Adipose‐Derived Stem Cells (ADSCs) Triggered for Fibroblastic Reticular Cell (FRC) Differentiation. A) i) Heatmap of the top 2000 expressed genes clustered into three groups using k‐means clustering. ii) GO Biological Processes pathways that were enriched in group two of heatmap, showing upregulated pathways in FRC_P1_ and FRC_P2_ samples compared to ADSC. B) PCA plot representing the distribution of FRC_P1_, FRC_P2_, and ADSC sequencing samples. C) Venn diagrams of the number of i) down‐ and ii) up‐regulated differentially expressed genes. D) Volcano plots of DEGs comparing i) FRC_P1_ versus ADSC, ii) FRC_P2_ versus ADSC, and iii) FRC_P1_ versus FRC_P2_. E) GO Biological Processes pathways and KEGG pathways that are enriched and upregulated in DEGs in FRC_P1_ and FRC_P2_ versus ADSC. Experiments were performed in triplicates with three different ADSC donors.

Through RNA sequencing (RNA‐seq), functional transcriptome analysis was performed to further understand the differentiation of ADSC toward FRC‐like cells. The overall heat map of differentially expressed genes distinctly shows that the gene expression profile between both FRC_P1_ and FRC_P2_, and ADSCs differs (**Figure** **2**A i). The top 20 upregulated biological pathways represented by genes in group two further show that FRC_P1_ and FRC_P2_ cells have upregulated biological pathways involved in cell migration, cytokine response, and general immune responses (Figure 2A ii). Principal component analysis (PCA) further demonstrates that the transcriptional profiles between FRC_P1_ and ADSCs are distinct, with FRC_P2_ having an intermediate profile closer to FRC_P1_, as expected (Figure 2B). Together, the hierarchical clustering of genes, PCA data, and upregulation of immune‐supportive biological pathways highlight the impact of TNF receptor and IL‐4 receptor signaling, alongside LT‐α‐mediated stimulation, in guiding ADSC differentiation toward an FRC‐like transcriptional profile.

We further analyzed both upregulated and downregulated differentially expressed genes (DEGs) using a false discovery rate (FDR) threshold of 0.1 and a minimum fold change of two. Using Venn diagrams to illustrate the shared genes between the three cell types, we found that there were 1011 downregulated and 1333 upregulated DEGs, with only 16 downregulated and 43 upregulated DEGs common across all three cell subsets (Figure 2C). Volcano plots were used to visualize the distribution of upregulated and downregulated DEGs among the different cell type comparisons, highlighting key gene signatures expected of FRCs (Figure 2D). This visualization indicates that there are more substantial gene differences between ADSCs and the FRC_P1_ and FRC_P2_ populations, with FRC_P1_ and FRC_P2_ being more similar to each other at the transcriptome level.

Upregulated pathway analysis of the DEGs in the respective cell subsets revealed that both FRC‐like cell subsets activated for FRC differentiation are enriched in genes related to immune responses, cell migration, and lymphocyte activation pathways, which are key functional features of FRCs (Figure 2E). However, due to the limited number of DEGs between FRC_P1_ and FRC_P2_, no detectable enriched pathways were identified to distinguish these two subsets. We further examined gene expression levels of ICAM1, PDPN, VCAM1, CD44, and Thy1, finding that their expression trends align with surface marker expression (representative flow cytometry analysis plots are shown in Figure S3A,B, Supporting Information), suggesting a direct correlation between gene and protein expression (Figure S3C,D, Supporting Information). This provides further assurance that our differentiated ADSC cells have been effectively converted into the various phenotypes of FRC‐like cells. The FRC_P1_ and FRC_P2_ populations are highly similar in their gene expression profiles and exhibit the functional characteristics necessary to support lymphoid tissue architecture and modulate immune responses.

### 3.3 FRC‐like Cells Exhibit Differential Remodeling of 3D Collagen Matrices

**Figure 3:**
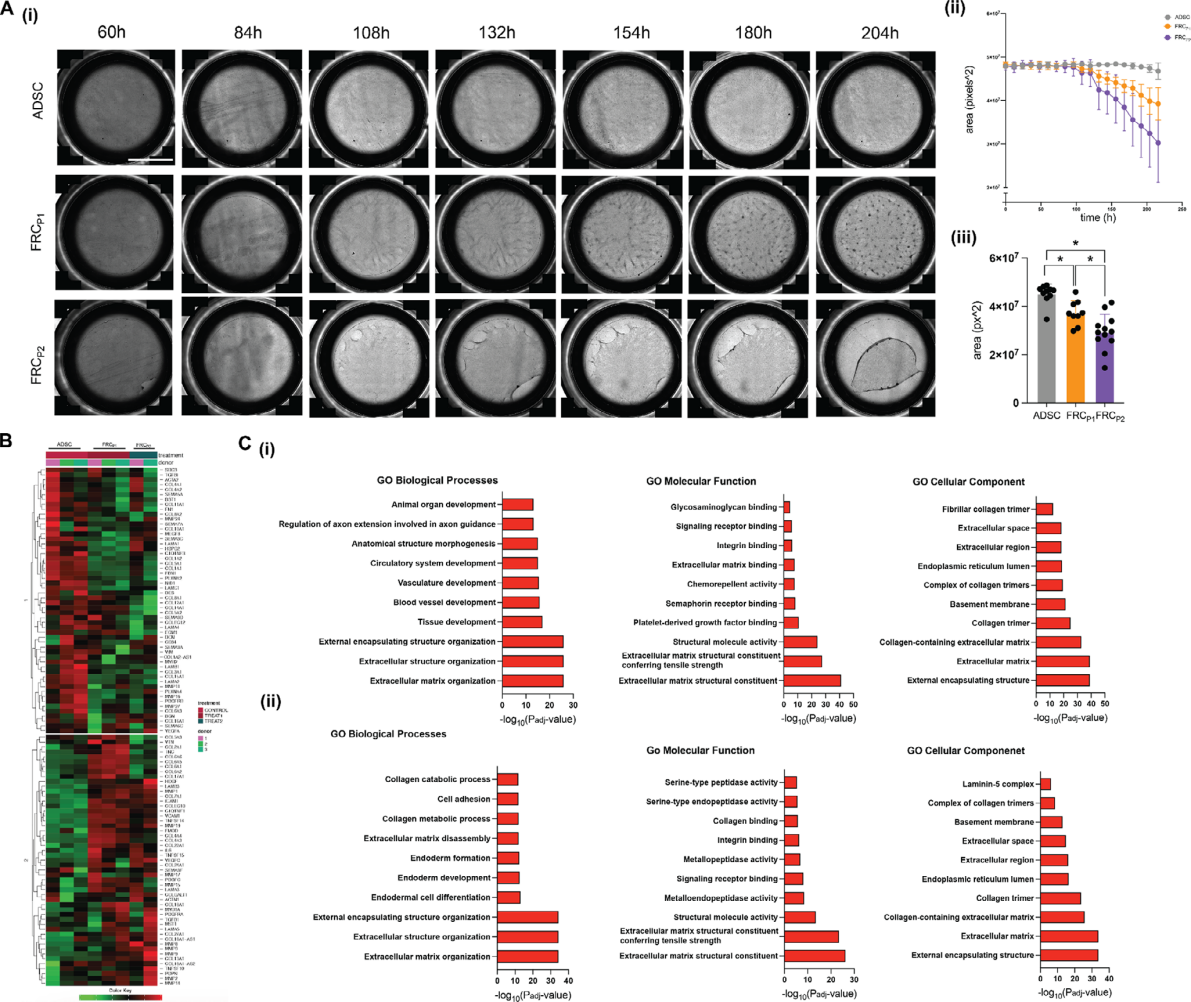
Embedded Fibroblastic Reticular‐like Cells (FRC) Remodel 3D Matrices. A) i) Representative images, taken every 24 h, showing full well images of ADSCs, FRC_P1_, and FRC_P2_ cells within collagen matrices during the 10‐day differentiation period. Images begin at 60 h when the first notable changes appear. Scale bar: 5 mm ii) Quantified change in matrix area over time for 10 days iii) Quantification of matrix area after 10 days of differentiation. B) Heatmap of extracellular matrix related genes clustered into two groups using k‐means clustering. C) Upregulated KEGG, GO Biological Processes, GO Molecular Function, and GO Cellular Component pathways of enriched genes in i) group 1 (ADSC vs FRC) and ii) group 2 (FRC vs ADSC) of the k‐means clustering heatmap.

One of the most notable changes observed during the FRC‐like cell differentiation was the remodeling of the 3D collagen matrices over time. Through whole sample imaging of collagen‐laden ADSCs undergoing differentiation, matrix contraction is observed in both FRC_P1_ and FRC_P2_ samplesstarts from the edges of the matrices (**Figure** **3**A i). The control ADSC samples remained relatively unchanged with little to no alterations in the matrix structure. Analysis over time illustrates that the area of the FRC_P2_ samples decreased at a faster rate than the FRC_P1_ samples (Figure 3A ii). Further quantification of the matrix area revealed that both FRC_P1_ and FRC_P2_ decreased significantly compared to ADSC controls, with the FRC_P2_ samples displaying a significantly greater contraction relative to FRC_P1_ (Figure 3A iii). The matrix contraction observed in the FRC‐like cells is likely linked to the expression of alpha‐smooth muscle actin (αSMA), a critical protein for the contraction of fibroblastic cells.^[^ ^40^ ^]^ Although αSMA is less commonly used in FRC phenotyping, its role in providing mechanical pre‐tension throughout the FRC network is essential for maintaining homeostatic conditions within lymphoid organs, as well as for regulating lymphoid organ size during inflammation.^[^ ^36^, ^38^ ^]^ Additionally, FRCs are known to play an important role in producing other ECM components and in matrix reorganization, which further supports the observed matrix remodeling during the differentiation process.^[^ ^38^ ^]^

To clarify the cellular and biological processes involved in this process, we conducted pathway analysis using Gene Ontology (GO) based on ECM related genes extracted from the RNA‐seq data. The k‐means clustered heatmap in Figure 3B revealed two distinct groups of gene clustering. Group 2 specifically contained genes that were upregulated in both FRC_P1_ and FRC_P2_ cells compared to ADSCs. The top 10 enriched GO Biological Processes pathways in this group (in decreasing fold change) included: extracellular matrix organization; extracellular structure organization; external structure organization; endoderm differentiation, development, formation; ECM disassembly; collagen metabolic process; cell adhesion; and collagen catabolic process (Figure 3C ii). These GO biological processes were associated with ECM assembly, disassembly, and organization, cell adhesion, and cellular differentiation. While the first three enriched processes of FRC‐like cells are also present in ADSCs, pathways related to the development of tissues, blood vessels, vasculature, and circulatory system were uniquely enriched for the ADSCs (Figure 3C i).

At the cellular level, GO Cellular Component analysis revealed that the top 10 enriched pathways were similar, with FRC‐like cells showing an upregulation of laminin‐5 complex and ADSCs having an upregulation of fibrillar collagen trimer. GO Molecular Function analysis identified differences in ECM‐related functions upregulated between our FRC‐like cells and ADSCs, were that of metallopeptidase and metalloendopeptidase activity (Figure 3C i,ii).

Collectively, these GO pathway findings align with our experimental intent (Figure 3C). We seeded pluripotent stem cells within lab‐engineered 3D collagen‐based hydrogels, where they attached and differentiated toward the endodermal lineage, a key component in lymphatic system development.^[^ ^41^ ^]^ As they progress through the differentiation, the evident remodeling seen from our daily temporal imaging would require FRC‐like cells to secrete metalloproteinases to break down the collagen scaffold, disassemble the ECM, and autologously secrete and assemble new ECM components. This process highlights the active role of FRC‐like cells in tissue remodeling and organization, which is critical for establishing the fibroblastic reticular network in TLOs and in our engineered 3D lymphoid tissue models.

### 3.4 Engineered Fibroblastic Reticular Networks Actively Support Immune Responses

**Figure 4:**
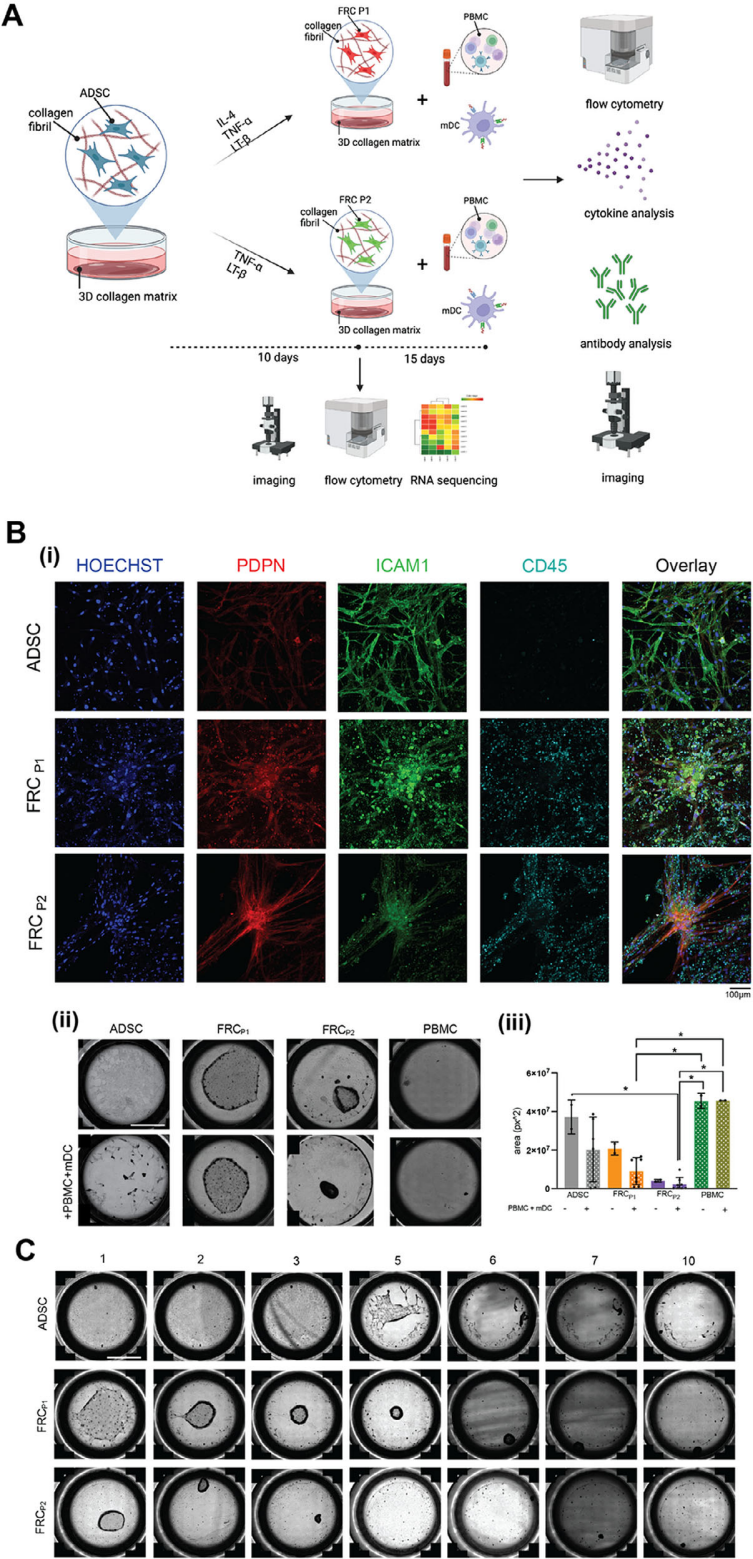
Co‐culture of Tissue Engineered Fibroblastic Reticular Cell (FRC) Networks with Peripheral Blood Mononuclear Cells (PBMCs) and Mature Dendritic Cells (mDCs) A) Experimental setup of the co‐culture of FRC‐like cells, differentiated from ADSCs with PBMCs and activated mDCs. Following the 15‐day co‐culture, analysis was performed using flow cytometry, imaging, and cytokine and antibody quantification. Created in https://BioRender.com B) i) Representative images taken at 20x on Olympus IX83 of ADSC, FRC_P1_, FRC_P2_ samples co‐cultured with PBMCs and mDCs. Samples were stained with Hoeschst (blue), PDPN (red), ICAM1 (green), and CD45 (cyan). ii) Representative images showing full‐well images of ADSCs, FRC_P1_, and FRC_P2_ cells, with and without PBMCs and mDCs, after 10 days of co‐culture in collagen matrices. Scale bar: 5 mm iii) Quantification of matrix area post co‐culture. Data are shown as mean +/‐ SD. * indicates significant difference at *p* ≤ 0.05. C) Representative images showing full well views of cultured ADSCs, FRC_P1_, and FRC_P2_ with PBMCs and mDCs, all within collagen matrices, taken at specific days post PBMCs and mDCs addition (indicated by numbers above the images). Scale bar: 5 mm.

Given that lymphoid tissues function as a convergence point for innate and adaptive immune system cross‐talk, and that the fibroblastic reticular network plays a role in modulating the immune responses, we co‐cultured our engineered lymphoid tissue with peripheral blood mononuclear cells (PBMCs) and mature dendritic cells (mDCs) that were primed with SARS‐CoV‐2 S1 spike protein to investigate these interactions (**Figure** **4**A). Flow cytometry analysis revealed increased levels of HLADR, CD80, and CD86, indicating that monocytes had successfully differentiated from immature dendritic cells (iDCs) into mDCs^[^ ^42^ ^]^ (Figure S4, Supporting Information). This maturation is essential for the subsequent recruitment of monocytes and lymphocytes in response to activated DCs, mimicking the early event in in vivo immune responses.

After allowing this co‐culture of PBMCs and mDCs to interact with FRC‐like cells within the 3D matrices for an additional 15 days, we performed endpoint analysis through imaging, flow cytometry, and enzyme‐linked immunosorbent assays (ELISAs) to test antibody, cytokine, and chemokine production (Figure 4A). Imaging reveals that PBMCs (stained with CD45) cluster around the larger FRC structures in the FRC_P1_ and FRC_P2_ samples but not around the ADSCs (Figure 4B i). Images of PBMC and mDC alone are seen in Figure S5 (Supporting Information) and show little to no clustering. These observations reflect the ability of the FRC network to recruit and spatially organize immune cells, a hallmark of lymphoid architecture. Interestingly, once PBMCs and mDCs are added, we observe increased contractility of the matrices compared to the conditions with only the FRC‐like cells (Figure 4B ii,iii). It is important to note that PBMCs alone and PBMCs cultured with mDCs do not result in any contraction of the matrices. This indicates that the enhanced contraction observed results from the biophysical interaction between the mixed culture and the tissue‐engineered fibroblastic reticulum. After 10 days, the contraction of the matrices reaches a level of no further observable changes. Hence, we have reported measurements and shown images taken up to 10 days post co‐culture, but performed all other analysis at the 15 day mark. Images taken for 10 days over the 15‐day protocol depict how the co‐culture samples contract over time (Figure 4C). Eventually, co‐cultures with FRC‐like cells form tiny spheroid‐like structures; FRC_P1_ shows a delay in formation, while the samples cultured with ADSCs appear to disintegrate the entire matrix. Together, these findings demonstrate that our engineered FRC‐like stromal tissues are not passive scaffolds but actively respond to immune cues, engage in immune–stromal communication, and support physical reorganization of the lymphoid‐like microenvironment in response to immune activation.

**Figure 5:**
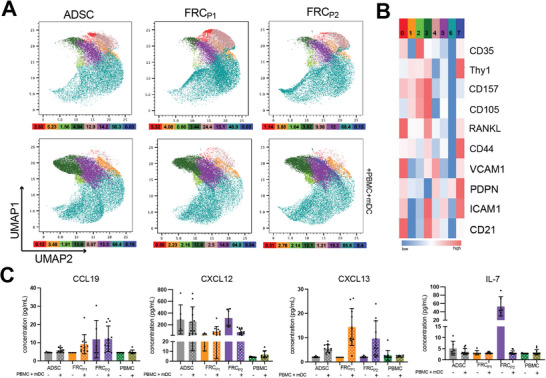
Change in Protein Expression of Tissue Engineered Fibroblastic Reticular Cells (FRCs) Following Co‐culture with Immune Cells Isolated from Peripheral Blood. A) Flow cytometry derived UMAP plot of live cell populations in ADSCs, FRC_P1_, and FRC_P2_ cells with and without PBMC and mDCs after 15 days of co‐culture. Each plot represents at least 6 concatenated samples using two distinct ADSC donors and two distinct PBMC donors. Values below each UMAP represent the percentage of each subpopulation within the treatment condition. Colors correspond to the subpopulation in the heatmaps. B) Heatmap of the expression of FRC markers in each subpopulation. Values are normalized within each column relative to the specific marker C) concentration of CCL19, CXCL12, CXCL13, and IL‐7 secreted by samples after 15 days co‐culture. Experiments were performed with at least 6 replicates. Two distinct ADSC donors and two distinct PBMC donors were used. Data are shown as mean +/‐ SD.

To assess the role of our tissue‐engineered FRC‐like cells in shaping immune microenvironments, we performed flow cytometry and cytokine profiling following 15‐day co‐culture with PBMCs and SARS‐CoV‐2 S1 protein–primed mDCs. Using UMAP plots, we visualized distinct clustering patterns indicating variations in the composition of the cell subpopulations under the different culture conditions (**Figure** **5**A). Representative figures of initial flow cytometry gating prior to UMAP processing can be found in Figure S6A (Supporting Information). The relative expression levels of the various markers were analyzed across the subpopulations, indicating the changes in marker expression with and without co‐culture with PBMCs and mDCs (Figure 5B). These patterns indicate shifts in subpopulation distribution and phenotypic remodeling in response to immune cell interaction. Since we anticipated that our spiked mDCs would communicate with the adaptive immune cells within the PBMC population and trigger an immune response, we were interested to see the involvement of our tissue engineered fibroblastic reticulum in aiding the process. Lymphoid organs are known to have a pivotal role in promoting the efficient activation and interaction of cognate lymphocytes.^[^ ^43^ ^]^ In particular, we aimed to determine if there were subsets of FRC‐like cells that could influence the compartmentalization of lymphocytes, a hallmark of structural patterning in secondary and tertiary lymphoid organs.

From our UMAP plots in Figure 5A and the corresponding heatmap in Figure 5B, we see that subpopulation 0, which is CD35+, CD157+, RANKL+, VCAM1+, PDPN+, and CD21+ could represent FDC‐like cells, which support B cell zones and is highest in our FRC_P1_ condition. Subpopulation 1 lacks defining stromal cell markers and could be a non‐specialized or undifferentiated fibroblast‐like population. Subpopulation 2, with high levels of CD35 and low levels of CD21 could represent pre‐FDC‐like cells, and this population has the highest levels in our FRC_P1_ co‐culture condition.^[^ ^34^ ^]^ Subpopulation 7, on the other hand, with low levels of CD21 and high levels of Thy1, CD44, VCAM1, ICAM1, and PDPN could be mature TRC‐like cells.^[^ ^34^ ^]^ Although this population consists of a small percentage overall, it is highest in our FRC_P2_ condition.

Because surface marker analysis alone is insufficient to deduce functional roles of cell populations, we analyzed cytokines and chemokines involved in lymphoid stroma functionality from the secretome of the different FRC types. The analysis showed elevated levels of CCL19, CXCL13, and IL‐7 in the FRC‐like cells compared to ADSC controls (Figure 5C; Figure S7, Supporting Information with visuals of statistical tests). CCL19, which is the key chemokine secreted by TRCs that binds to CCR7 and attracts T cells and DCs to T cell zones in lymphoid tissues, was secreted significantly higher in FRC_P2_ (11.91 ± 10.26 pg mL^−1^) relative to the level produced by ADSCs or FRC_P1_ samples alone (4.62 ± 0.24 and 4.52 pg mL^−1^). Upon the addition of PBMCs and mDCs, the levels in the FRC_P1_ samples increased slightly (9.17 ± 5.2 pg mL^−1^), but the FRC_P2_ co‐culture condition still produced the highest concentration of CCL19 (average of 12 ± 7.02 pg mL^−1^). This is consistent with the UMAP data above showing the highest levels of subpopulation 7, thought to be TRC‐like cells, in the FRC_P2_ condition. In the control samples, the addition of PBMCs and mDCs significantly increased the concentration of CCL19 to almost 6 pg mL^−1^ compared to 4.6 pg mL^−1^ produced by ADSCs alone. When PBMCs are cultured alone or with the addition of mDCs, there is no significant increase in the amount of CCL19 produced relative to ADSCs alone. CXCL12 has been shown to support DC and lymphocyte retention in lymphoid tissues and is naturally secreted by ADSCs.^[^ ^44^ ^]^ We see that it is produced at high levels by all conditions except PBMCs and PBMC with mDCs. Secreted CXCL12 in both FRC_P2_ (319 ± 150.4 pg mL^−1^) and ADSC (291.6 ± 248.8 pg mL^−1^) is significantly higher than FRC_P1_ condition (93.59 ± 115.3 pg mL^−1^), possibly due to IL‐4‐mediated downregulation.^[^ ^45^ ^]^ CXCL13 is produced primarily by FDCs and is critical for B follicle formation.^[^ ^34^ ^]^ Our data shows that this chemokine is produced at significantly higher concentration in all the co‐culture conditions compared the their single culture counterparts, with the exception of the PBMC and PBMC with mDC controls. The co‐culture of FRC_P1_ cells with PBMC and mDC produces the highest overall amounts of CXCL13 at an average of 14.4 ± 7.64 pg mL^−1^. This is in accordance with the UMAP data in Figure 5A, showing that the FRC_P1_ condition has the highest levels of FDC‐like cells represented by subpopulation 0. IL‐7, a critical survival cytokine for T cells that is produced primarily by TRCs, is secreted at a 10 times higher concentration by FRC_P2_ cells (53.24 ± 23.24 pg mL^−1^) than any other condition. When PBMCs and mDCs were added to the FRC_P2_ sample, the levels of IL‐7 decreased significantly (to 3.21 ± 0.79 pg mL^−1^), indicating that perhaps this cytokine is being bound by the T cells in the culture. Again, this data is in agreement with the UMAP indication of FRC_P2_ samples having the highest amount of TRC‐like cells.^[^ ^36^ ^]^ This data is an indication that our FRC‐like cells have the necessary cytokine cues to orchestrate immune cell activation.

**Figure 6:**
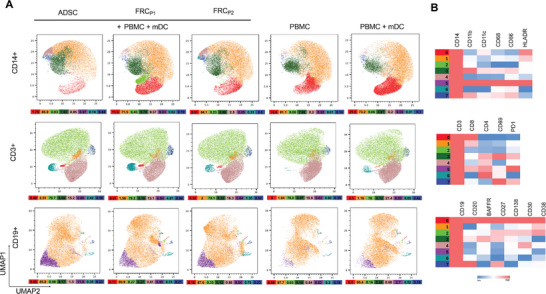
Protein Expression of Peripheral Blood Immune Cells (PBMCs) Following 15 Day Co‐culture with Tissue Engineered Fibroblastic Reticular‐like Cells (FRCs). A) Flow cytometry‐derived UMAP plot of populations in live cells within CD14+, CD3+, and CD19+ cells after 15 days of co‐culture. Each plot represents at least 10 concatenated samples using two distinct ADSC donors and two distinct PBMC donors. Values below each UMAP represent the percentage of each subpopulation within the treatment condition. Colors correspond to the subpopulation in the heatmaps. B) Heatmaps of the expression of relative cell surface markers in each cell population. Values are normalized within each column relative to the specific marker.

If the FRC‐orchestrated immune landscape successfully establishes the foundation for initiation of an immune response, we would expect to see appropriate immune cell populations being activated. To evaluate this, we analyzed CD14+ monocytes, CD3+ T cells, and CD19+ B cells from the co‐culture with our tissue engineered fibroblastic reticulum (**Figure** **6**). CD14+ cells represent the monocytes present in PBMCs as well as the mDCs that were primed prior to the co‐culture experiments. Representative figures of initial flow cytometry gating prior to UMAP processing can be found in Figure S6B (Supporting Information). We analyzed several markers within this subset of cells, including CD11b, CD11c, CD86, CD68, and HLADR. In order to invoke the adaptive immune response effectively, mDCs must express maturation markers HLADR along with the costimulatory protein CD86, on their surface, for the activation and cross presentation to lymphocytes.^[^ ^42^ ^]^ We observed co‐expression of these markers in subpopulations 3 and 5. Both these subpopulations were double‐positive for CD11b and CD11c, which are markers of dendritic cells.^[^ ^46^ ^]^ The difference in HLADR and CD86 expression levels between these two subpopulations suggests varying levels of maturity, with subpopulation 5 representing a more mature population of DCs than subpopulation 3. This may indicate that subpopulation 5 includes the mDCs matured prior to co‐culture, as well as some CD14+ monocytes from the PBMC population that underwent differentiation into DCs during co‐culture. On the other hand, subpopulation 3 could predominantly consist of mDCs that were matured prior to co‐culture. These findings affirm the functional maturation of monocytes within the FRC‐driven immunological landscape, particularly in the FRC_P1_ co‐culture condition, which has the highest percentage of subpopulation 3.

Subpopulation 7 exhibited high expression of all the analyzed markers, except for HLADR and CD11c, which may indicate that it represents M2 macrophages in the culture, since it expresses the pan‐macrophage marker CD68 (Figure 6B).^[^ ^47^ ^]^ Interestingly, with the exception of subpopulations 1, 4, and 7, all other subpopulations are present at higher percentages in FRC_P1_ as compared to FRC_P2_ co‐cultures (Figure 6A). Despite the differences, there are indeed populations of cells in both FRC_P1_ and FRC_P2_ co‐cultures that are capable of antigen presentation and therefore contribute to the initiation of immune responses

**Figure 7:**
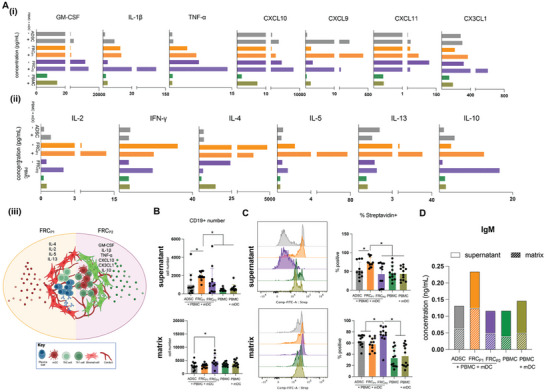
Secretome Analysis from Tissue Engineered Fibroblastic Reticulum. A) i, ii) Concentration of cytokines produced by fibroblastic reticular like cells (FRC‐like) samples after 15‐day co‐culture. A) iii) Schematic of potential microenvironments created by FRC_P1_ and FRC_P2_ subtypes. FRC_P1_ supports plasma cell formation and Th2‐skewed environment. FRC_P2_ supports DCs and Th1‐skewed environment. Created in https://BioRender.com B) Number of live CD19+ cells in each population in supernatant and matrices. C) Representative histograms of streptavidin+ cells in each condition and quantification of the percentage of streptavidin+ cells within live CD19+ population. D) Concentration of SARS‐CoV‐2 S1 protein‐specific IgM in supernatant and matrix of all conditions. All experiments were performed with at least 6 replicates. Two distinct ADSC donors and two distinct PBMC donors were used in all experiments. Data are shown as mean +/‐ SD. * indicates a significant difference of *p* ≤ 0.05.

To further assess the capacity of the engineered fibroblastic reticular networks to support myeloid activation and antigen presentation, we measured key cytokines and chemokines involved in monocyte‐to‐DC differentiation and Th1 cell recruitment (**Figure** **7**A i). Raw concentration values (mean±SD) of all cytokines are found in Figure S8 (Supporting Information). GM‐CSF, a cytokine critical for promoting monocyte‐to‐DC differentiation, was markedly elevated in FRC_P2_ monocultures (10676 ± 10 219 pg mL^−1^) and further increased in FRC_P2_ co‐cultured with PBMCs and mature dendritic cells (12920 ± 8777 pg mL^−1^), representing a 1.2‐fold increase relative to the monoculture and almost 5‐fold increase to the ADSC co‐culture controls. This indicates a strong stromal capacity to direct myeloid differentiation (Figure 7A).^[^ ^48^ ^]^ Similarly, IL‐1β, a marker of inflammasome activation, was highest in FRC_P2_ co‐cultures (≈112 ± 117.9 pg mL^−1^) with a 9‐fold increase compared to ADSC and almost 6‐fold increase compared to FRC_P1_ co‐cultures, suggesting that FRC_P2_ may promote a more pro‐inflammatory environment conducive to initiating antigen presentation.^[^ ^49^ ^]^ TNF‐α, a cytokine involved in DC licensing and local inflammation, was significantly upregulated in all FRC‐containing conditions, with the highest levels observed in FRC_P2_ co‐cultures (14.01 ± 5.8 pg mL^−1^), reinforcing the role of the FRC matrix in shaping immune activation niches.^[^ ^50^ ^]^

A Th1‐supportive environment in the FRC_P2_ conditions was indicated by elevated secretion of CXCR3 ligands, CXCL10 and CXCL11, which mediate T cell recruitment.^[^ ^51^ ^]^ CXCL10 levels were highest in FRC_P2_ co‐culture (7969 ± 3030 pg mL^−1^), followed by FRC_P2_ monocultures (3909 ± 3339 pg mL^−1^), indicating an IFN‐γ–associated Th1 chemotactic axis.^[^ ^52^ ^]^ Interestingly, CXCL9, also involved in T cell recruitment, peaked in FRC_P1_ co‐cultures (422 ± 281.3 pg mL^−1^), suggesting differential chemokine signaling between the two FRC subtypes.^[^ ^53^ ^]^ CXCL11 was most strongly expressed in FRC_P2_ monocultures (115 pg mL^−1^), consistent with a TRC‐like role in coordinating T cell entry. Finally, CX3CL1, which attracts both monocytes and effector T cells to inflamed tissues, was significantly elevated in FRC_P2_ co‐cultures (576 ± 284.9 pg mL^−1^), highlighting its potential role in shaping inflamed stromal microenvironments.^[^ ^54^ ^]^ Together, these data demonstrate that FRC_P2_ creates a highly immune‐stimulatory and antigen‐presenting niche resembling lymph node T cell zones.

The adaptive immune system is initiated through an immunological synapse formed by antigen presenting cells, such as mDCs, which present antigens to T and B cells to trigger responses. To further investigate this response, we analyzed the subpopulations of CD3+ T cells and CD19+ B cells. For T cells, we analyzed CD4+ helper T cells and CD8+ cytotoxic T cells for CD69, an early activation marker, and PD1, a T cell exhaustion marker, or also an early activation marker.^[^ ^55^ ^]^ Within CD3+ cells, a pan T‐cell marker, we identified several important subpopulations. Subpopulations 3, 4, and 6 are all CD4+ cells, with subpopulation 6 being CD69+, and subpopulation 3 being CD69+ and PD‐1+, indicating an activated state. On the other hand, subpopulation 4 had low expression of both markers, suggesting an inactivated or resting state. The activated helper T cells subpopulations have similar percentages in both the FRC_P1_ and FRC_P2_ co‐culture conditions (Figure 6A).

Subpopulations 0 and 1 were both CD8+ T cells. While subpopulation 1 expressed slightly higher levels of PD‐1, the levels of CD69 and PD‐1 in both CD8+ populations indicated non‐activated, and therefore non exhausted T cells. Subpopulation 2 and 7 consisted of naive or CD4‐ CD8‐ double negative (DN) T cells, and subpopulation 5 comprised of either both CD4+ and CD8+ cells or CD4+ CD8+ double positive (DP) cells. The DN and DP cells are rare and unique populations that are still controversial, and their exact functions are not yet well established.^[^ ^56^, ^57^, ^58^ ^]^

To evaluate the influence of engineered FRC networks on adaptive immunity, we profiled cytokines associated with T cell activation and helper T cell subset polarization (Figure 7A ii). IL‐2, a key survival factor for T cells, was strongly elevated in FRC_P1_ co‐cultures (12.31 ± 11.45 pg mL^−1^) and moderately in FRC_P2_ co‐cultures (2.04 ± 1.9 pg mL^−1^), compared to minimal levels in PBMCs or ADSC conditions.^[^ ^59^ ^]^ This suggests a robust FRC‐driven environment for T cell proliferation, particularly in the FRC_P1_ network. IFN‐γ, indicative of Th1 polarization, was highest in FRC_P1_ monocultures (32.1 ± 18.14 pg mL^−1^) and elevated across FRC_P1_ and FRC_P2_ co‐culture conditions (≈18–19 pg mL^−1^), supporting a potential role for both FRC_P1_ and FRC_P2_ in promoting Th1‐skewed immunity. However, subsequent analysis of other cytokines shows support for FRC_P1_ being a Th2 supporting environment. Strikingly, IL‐4 levels were extremely high in FRC_P1_ monocultures and co‐cultures (4676 ± 1913 and 2555 ± 2364 pg mL^−1^, respectively), whereas FRC_P2_ produced significantly less IL‐4. This Th2‐associated cytokine profile was further supported by elevated IL‐5, IL‐13, and IL‐10 levels in FRC_P1_ + PBMC + mDC co‐cultures, reaching 73.9 ± 93.87, 30.18 ± 38.09, and 12.28 ± 7.3 pg mL^−1^ respectively.^[^ ^60^ ^]^ These findings indicate a strong Th2 bias within the FRC_P1_ network, conducive with the data seen from the UMAPs and chemokine profiles in Figures 5 and 6.^[^ ^61^ ^]^ In contrast, FRC_P2_ conditions produced lower levels of IL‐4 and IL‐13, but showed high IL‐10 in co‐cultures (16.52 ± 12.15 pg mL^−1^), suggesting an immunoregulatory or transitional state rather than a polarized Th2 response. Together, these data suggest that FRC_P1_ strongly promotes Th2‐like polarization and robust T cell proliferation, while FRC_P2_ favors Th1‐associated responses and immunomodulatory cytokine production. This divergence in cytokine output reflects the functional specialization of the FRC subtypes in shaping the adaptive immune landscape, with FRC_P1_ supporting humoral‐oriented immunity and FRC_P2_ providing structural support for cell‐mediated immunity and immune regulation.

The adaptive immune system also consists of the humoral immune response, where highly specific immunoglobulins (or antibodies) are generated by B cells. These antibodies then bind to antigens, abrogate their infectious nature, and contribute to the clearance of pathogens. B cells are critical in this process and undergo an interactive affinity maturation process that eventually results in plasma cells responsible for antibody production.^[^ ^62^ ^]^ In our analysis of CD19+ B cells, subpopulation 7, expressing CD19+CD20+CD27+ and low levels of CD38, likely represents follicular B cells (Figure 6A).^[^ ^63^ ^]^ These cells recirculate around the body, but more importantly, in the context of this study, home to lymphoid tissues and participate in the T cell‐dependent humoral response.^[^ ^64^ ^]^ In order for plasma cells to be generated, follicular B cells have to enter a germinal center, specialized structures within lymphoid tissues essential for affinity maturation and that require the support of FRCs.^[^ ^65^ ^]^ The percentage of follicular B cells within CD19+ cells are found to be increased in co‐cultures with stroma cells compared to the PBMC and mDC control groups. Subpopulation 2, which is CD27+ and CD38+ with low levels of CD20, may correspond to plasma cells. Notably, subpopulation 2 expressed CD138, a marker of fully differentiated plasma cells, which is consistent with cells actively producing antibodies.^[^ ^66^ ^]^ The percentage of these cells in the co‐cultures are higher in the FRC_P1_ and FRC_P2_ samples compared to the other conditions (Figure 6A).

To evaluate the capacity of the engineered FRC networks to support antigen‐specific B cell activation and antibody production, we assessed the presence of SARS‐CoV‐2 S1 protein‐specific B cell receptors (BCRs) and the generation of SARS‐CoV‐2 S1 protein‐specific antibodies in both the culture supernatant and the embedded collagen matrix. Using biotinylated SARS‐CoV‐2 S1 protein and FITC‐labeled streptavidin, we were able to quantify antigen‐specific BCRs in our culture by flow cytometry. We first quantified the number of live CD19⁺ B cells and subsequently identified the proportion of streptavidin‐positive cells, indicating the presence of antigen‐specific BCRs on their surface (Figure 7B,C). Representative histograms depicting this population can be seen in Figure 7C. B cells, and in particular mature plasma cells, are known to egress from lymph nodes and lymphoid tissues to other sites such as bone marrow.^[^ ^67^ ^]^ Because of this, we tested both the cells that were present in the supernatant of the samples as well as those that remained embedded in the matrix. In the supernatant, both the total number of live CD19⁺ B cells and the percentage of streptavidin‐binding B cells were significantly higher in the FRC_P1_ co‐cultures compared to ADSC, FRC_P2_, PBMC, and PBMC with mDC conditions (Figure 7B,C). However, in the matrix, these numbers were higher in FRC_P2_ co‐cultured samples, which could be due to the fact that the matured, and specific, plasma cells in the FRC_P1_ condition have egressed from the matrix into the supernatant, or that there was a lack of retention by the stromal network. This data suggests that FRC_P1_ may provide a niche more conducive to B cell localization and activation within the stromal architecture.

Consistent with this, ELISA quantification of SARS‐CoV‐2 S1 protein‐specific IgM antibodies revealed significantly elevated levels in both supernatant and matrices of FRC_P1_ cultures, compared to controls and FRC_P2_ (Figure 7D). Raw concentration values for each sample can be found in Figure S9A (Supporting Information). Notably, measurable antibody levels were also detected within the collagen matrix, indicating active local antibody production or retention within the tissue‐engineered niche. Studies have shown that antibodies are likely to get stuck or be retained within collagen matrices, making them harder to detect in supernatant alone.^[^ ^19^ ^]^ Although SARS‐CoV‐2 S1‐specific IgG concentrations were consistently higher than IgM across all co‐culture conditions, the levels of IgG remained relatively uniform, showing only modest variation between FRC‐containing and PBMC‐only groups (Figure S9B, Supporting Information). This may be due to the fact that IgG production typically requires germinal center formation, affinity maturation, and class‐switch recombination, which are processes that take longer to develop than the early IgM response.^[^ ^68^ ^]^ Given the shorter duration of the culture period, it is likely that the observed IgG levels represent either background or early class‐switched responses that had not yet fully matured. These results suggest that FRC_P1_ may preferentially enhance early B cell activation and specific IgM secretion, whereas IgG responses may require extended culture durations or additional maturation signals not yet present in this in vitro model.

## 4 General Discussion and Conclusion

In this study, we achieved our central aim of developing an in vitro lymphoid tissue model capable of producing antigen‐specific antibodies by differentiating ADSCs into FRC‐like cells and incorporating PBMCs and antigen‐primed mature dendritic cells. This represents a significant step toward generating biomimetic immune environments ex vivo. While the precise molecular mechanisms underlying FRC‐like differentiation and immune support remain to be fully elucidated, our results demonstrate that these engineered tissues are functionally competent and can support adaptive immune responses. Furthermore, the use of ADSCs as a starting material highlights the potential of this system to be personalized, offering a scalable strategy to generate patient‐specific lymphoid models for immunotherapy or vaccine.

Our results demonstrated that ADSCs can be successfully differentiated into FRC‐like cells using two distinct cytokine protocols (FRC_P1_ and FRC_P2_) within a 3D collagen matrix. Both protocols yielded FRC‐like cells with distinct phenotypes as revealed by microscopy, flow cytometry, and RNA‐sequencing analyses (Figures 1, 2, and 3). The FRC_P1_ cells formed more pronounced clusters and exhibited less matrix contraction compared to the FRC_P2_ cells that had an elongated morphology and contributed to extensive matrix contraction. This was ascertained by higher expressions of PDPN in FRC_P2_, a protein that drives actomyosin‐mediated contraction.^[^ ^69^ ^]^ Immunofluorescence staining for PDPN and ICAM1 were minimally expressed in ADSCs but strongly upregulated in both FRC_P1_ and FRC_P2_ populations, with FRC_P2_ showing particularly high PDPN expression. This staining not only confirmed the acquisition of canonical FRC markers, and corroborated quantitative findings obtained through flow cytometry, but also revealed their spatial distribution within multicellular clusters in the 3D collagen matrix. Importantly, the higher PDPN levels observed in FRC_P2_ are consistent with their enhanced contractile phenotype and matrix remodeling capacity, features typically associated with T‐zone reticular cells.^[^ ^70^ ^]^ In contrast, FRC_P1_ displayed a comparatively lower PDPN profile but shared ICAM1 upregulation, in line with their skewing toward a B cell–supportive, FDC‐like phenotype.^[^ ^71^ ^]^ Together, these findings provide additional validation of the FRC‐like identity of the differentiated cells and strengthen the conclusion that FRC_P1_ and FRC_P2_ represent functionally distinct stromal subsets with specialized roles in immune regulation.

Transcriptomic analysis revealed that both FRC_P1_ and FRC_P2_ cells upregulated genes involved in immune response pathways, cell migration, and ECM remodeling compared to the ADSC controls. FRC_P1_ and FRC_P2_ shared a high degree of transcriptomic similarity, particularly in their enrichment of immune‐related pathways. Interestingly, the distinct contraction patterns observed in the matrices embedded with FRC_P1_ and FRC_P2_ cells correlated with differences in ECM‐related gene expression compared to ADSCs, highlighting the role of FRC‐like cells in matrix remodeling. This suggests that FRC‐like cells, particularly FRC_P2_, possess a strong capacity for modifying their surrounding ECM, a critical feature of lymphoid organs where FRCs support the migration of immune cells and the organization of immune responses.^[^ ^39^, ^69^ ^]^ In line with our RNA sequencing data showing enrichment of pathways such as innate immune response, chemokine signaling, and response to virus pathways, we functionally validated these findings through cytokine and chemokine profiling, demonstrating robust secretion of CCL19, CXCL13, IL‐7, GM‐CSF, IL‐4, and IFN‐γ, thereby confirming that the differentiated FRC‐like cells not only display a transcriptional FRC signature but also engage in immune‐supportive functions at the protein level.

Although both FRC‐like cell subsets were similar at a transcriptome level, we see differences in their morphology and matrix remodeling capabilities and therefore assumed that they would provide different functions within the lymphoid model. To test this hypothesis, we co‐cultured each of these cells with the mix of PBMCs and SARS‐Cov‐2 S1 protein primed mDCs. Notably, the addition of immune cells led to increased matrix contraction, with FRC_P2_ contracting at a faster rate than FRC_P1_ cells (Figure 4). This increased contraction is particularly important in lymphoid organs whereby the anatomical structure is in a pre‐stressed state to cater for the expansion of lymphocytes during the inflammation process.^[^ ^72^ ^]^

From flow cytometry and through dimensionality reduction via UMAP analysis, it was revealed that distinct changes occurred in the surface marker expression of both FRC‐like cells and immune cells during co‐culture. Moreover, our co‐culture experiments demonstrated that FRC‐like cells influenced the differentiation and activation of monocytes, T‐, and B‐cells within PBMC populations in addition to the production of measurable amounts of immune mediating chemokines and cytokines (Figures 6 and 7). Studies have shown that within lymphoid organs, the T cell zone contains more Th1‐specific cytokines such as IL‐2 and IFN‐γ, whereas B cell zones express more Th2 cytokines such as IL‐4.^[^ ^73^ ^]^ From our data, we see similar results that lead us to believe that our FRC_P1_ samples create a more B cell friendly zone due to their high secretion of IL‐4, IL‐5, IL‐13, and CXCL13 (Figures 5 and 7). In addition to the FDC‐like cells observed from our UMAPs, IL‐4 has been shown to amplify germinal center formation and response, further supporting the hypothesis that FRC_P1_ differentiation skews stem cells into a B cell supporting subset of stromal cells.^[^ ^74^ ^]^ While both FRC_P1_ and FRC_P2_ secrete IL‐2 and IFN‐γ, the higher levels of CCL19 and IL‐7 present in the FRC_P2_ samples, in addition to the higher levels of TRC‐like cells, CXCL10, and CX3CL1, lead us to postulate that this condition generates a microenvironment more supportive of T cells (Figures 5 and 7).^[^ ^75^ ^]^

B cells, and in particular mature plasma cells, are known to egress from lymph nodes and lymphoid tissues to other sites such as bone marrow. This was supported by the fact that in our FRC_P1_ system, we observed higher numbers of B cells with antigen‐specific BCRs in the supernatant compared to residing in the matrix (Figure 7). In addition, our FRC_P1_ condition produced significantly higher levels of antigen‐specific IgM antibodies, adding to the notion that mature plasma cells were produced by this condition. However, our current assay does not directly measure sphingosine‐1‐phosphate (S1P)‐mediated egress but rather the presence of cells outside the matrix, which could reflect a lack of retention within the stromal network. Future studies incorporating direct quantification of S1P signaling or live‐cell tracking will be required to distinguish between true egress and reduced retention.^[^ ^76^ ^]^

A noteworthy observation was the impact of FRC‐like cells on immune responses despite the use of ADSCs and PBMCs from different donors. While baseline co‐cultures of ADSCs with PBMCs showed immune cell activation and cytokine secretion, the introduction of differentiated FRC‐like cells (FRC_P1_ and FRC_P2_) into the system resulted in markedly enhanced immune responses. This contrast highlights the unique immunological influence of the FRC network, as opposed to undifferentiated ADSCs, demonstrating that the immunological outcomes observed in our study are not merely the result of donor‐specific variability but are instead driven by the specialized function of the differentiated FRC‐like cells. It is important to emphasize that ADSCs have inherently low immunogenicity, which makes them an ideal candidate for generating FRC‐like cells, even in allogeneic settings.^[^ ^77^ ^]^ Unlike other stromal cell sources, ADSCs can evade immune rejection due to their low expression of major histocompatibility complex (MHC) class II molecules and co‐stimulatory markers.^[^ ^78^ ^]^ Our CD8+ cells from the co‐cultures were not activated further emphasizing the low immunogenicity of ADSCs (Figure 6). This characteristic minimizes the risk of immune activation when co‐cultured with PBMCs from unrelated donors. However, the differentiation of ADSCs into FRC‐like cells modifies their immune properties, enabling them to engage in immune cell crosstalk and contribute to a functional lymphoid‐like microenvironment. This finding underscores the robustness and physiological relevance of our model. It demonstrates that FRC‐like cells derived from ADSCs possess the functional capacity to mimic the native FRC network, even in a heterologous system. Future work will expand the donor pool and, when possible, utilize donor matched ADSCs and PBMCs to strengthen translational relevance.

A caveat in our study is the interpretation of subtypes of FRC‐like cell types. The classification of the subtypes was entirely based on RNA sequencing, protein expression, and secretome population‐based measurements. In reality, there could be FRC_P1_ cells in FRC_P2_ classified cell culture, albeit a relatively small percentage, and vice versa. The spatial distribution of each subtype of FRC‐like cell types within our classified FRC_P1_ or FRC_P2_ is unknown in our investigations, but could possibly be assessed using advanced techniques like single cell or spatial transcriptomics, which have inherent resolution and sensitivity issues and prohibitive cost considerations. Additionally, future work such as evaluating the capability of these FRC‐like cells to present antigen and assessing peripheral tissue antigen expression could strengthen the interpretation of these subtypes.^[^ ^79^ ^]^ In addition, optimizing the differentiation protocols could lead to an increased percentage of certain stromal subpopulations that could result in a more robust functional outcome. Even so, we are able to produce functional lymphoid tissue and the ability to generate cytokines, support T and B cell activation, and drive specific antibody production validates the effectiveness and value of our 3D model for expansive investigation into intricate mechanistic immune responses within lymphoid tissues. Even more importantly, this also has transformative implications for future applications, including the development of personalized immunotherapies or vaccine testing platforms, where ADSCs can be utilized as a scalable and minimally invasive cell source due to their wide availability from adipose tissue, which can further enhance their feasibility for precise clinical translation.^[^ ^80^ ^]^ The differences in our two FRC‐like subtypes (FRC_P1_ and FRC_P2_) suggest that the cytokine composition used for differentiation can significantly influence the structural properties and immunological behavior of the resulting FRC‐like cells, an observation that could be leveraged to tailor the lymphoid model for specific applications, such as using FRC_P1_ for antibody production or vaccine testing and using FRC_P2_ for more T cell activation responses.

**Figure 8:**
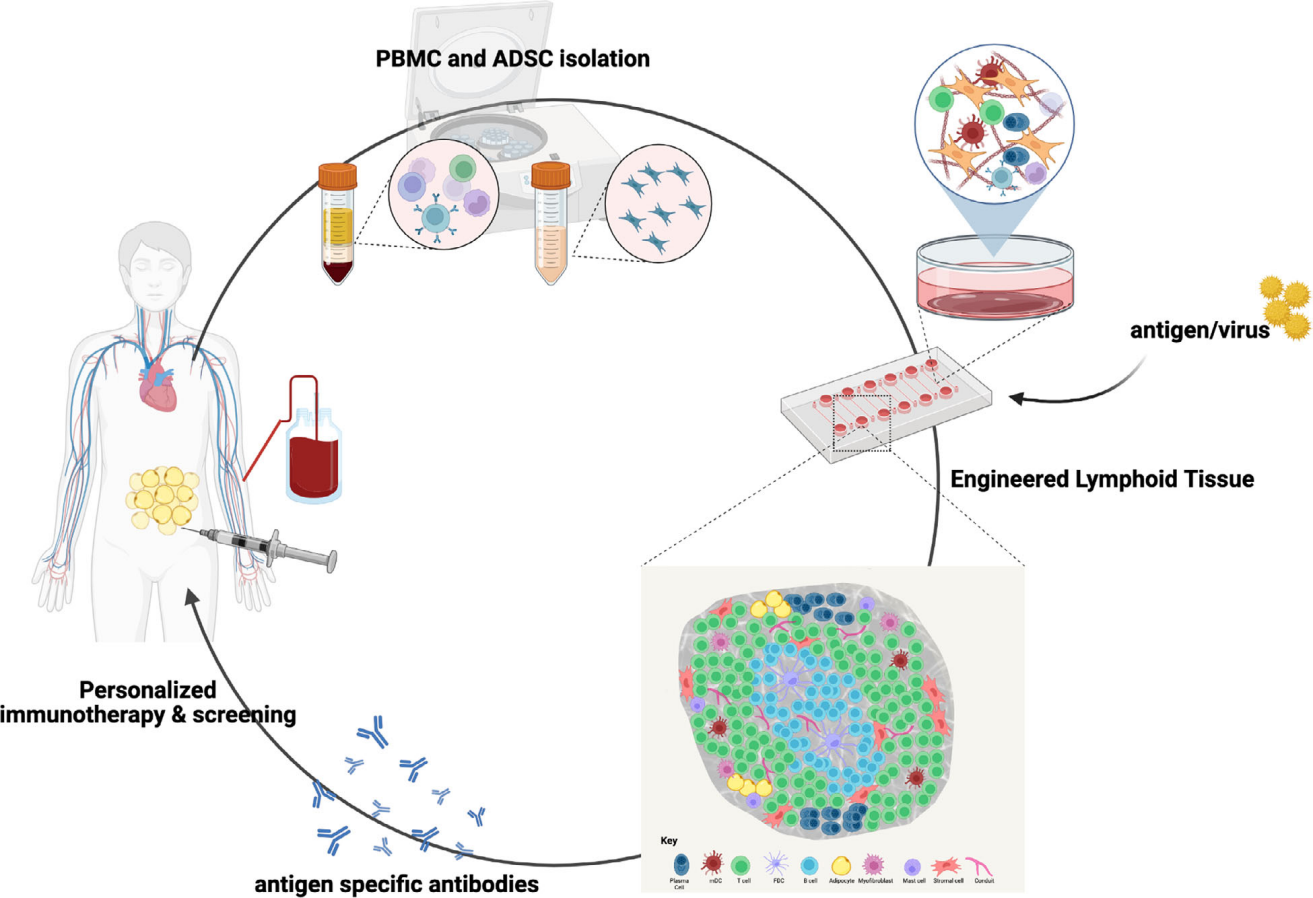
Potential for personalized therapy using engineered lymphoid tissue model on a microfluidic chip. Antigen‐specific antibodies can be produced using ADSCs and PBMCs from a donor, resulting in personalized therapy. Created in https://BioRender.com.

While our model provides important insights into FRC differentiation and immune cell interactions, there are still limitations to be addressed in the future. Further refinement of the differentiation protocols and a deeper understanding of the molecular mechanisms driving FRC function will enhance the model's capacity to fully replicate the complexity of a lymphoid organ microenvironment. Additionally, the inclusion of other stromal cell types, for example blood and lymphatic endothelial cells, could further improve the models physiological relevance. Future work will also focus on improving the scalability of the organotypic model, enhancing its ability to generate specific immune responses to various antigens, and adapting it for personalized immunotherapy applications, since adipose derived stem cell isolation is minimally invasive (**Figure** **8**).^[^ ^81^ ^]^ Exposing the organotypic lymphoid tissue to flow induced shear stress within microfluidic chips, mimicking enhanced fluid flow during inflammation, has also been shown to enhance T‐cell and plasma cell response.^[^ ^19^, ^82^, ^83^ ^]^ The evidence throughout this study is endorsing and that our 3D lymphoid tissue model can effectively recapitulate aspects of cellular recruiting, directing, and subsequent immune signaling. The overall detection of specific cytokines and antibodies suggests that this system could be used for studying immune responses against novel antigens, evaluating vaccine efficacy, or exploring the dysregulation of immune processes in diseases such as autoimmunity.

## Conflict of Interest

The authors declare no conflict of interest.

## Author Contributions

M.E. and J.T. contributed to conceptualization. Formal analysis was performed by M.E. and S.K. Funding acquisition was carried out by J.T. Experimental investigation was done by M.E. and S.K. Methodology was developed by M.E. Supervision was carried out by J.T. Visualization was performed by M.E. and S.K. Writing of the original draft was done by M.E. Writing, reviewing, and editing were done by S.K. and J.T. All authors have read and agreed to the published version of the manuscript.

## Supporting information



Supporting Information

## Data Availability

The data that support the findings of this study are available from the corresponding author upon reasonable request.
