# Supplementary material for: Engineering Adaptive Immunity in 3D: A Patient‐Specific Lymphoid Model Using Stromal Networks and Peripheral Blood Mononuclear Cells
Source: Adv Sci (Weinh). 2025 Dec 16;13(12):e13245. doi: 10.1002/advs.202513245 (PMC12948259; doi:10.1002/advs.202513245)
Supplement: Supplementary file 1 — Supporting Information [file ADVS-13-e13245-s001.docx]

**Supplementary information**

Mei ElGindi^1^, Shaza Karaman^1^, Jeremy Teo^1,2*^

1. Laboratory for Immuno Bioengineering Research and Applications, Division of Engineering, New York University Abu Dhabi, Abu Dhabi POBox 129188, United Arab Emirates; me95@nyu.edu (M.E.); [shaza.karaman@nyu.edu](mailto:shaza.karaman@nyu.edu) (S.K.); jeremy.teo@nyu.edu (J.T.)

2. Department of Mechanical and Biomedical Engineering, New York University, Brooklyn, NY 11201, USA

*****Correspondence: jeremy.teo@nyu.edu; Tel.: +971-2-6286689

**Supplementary Table 1**: Details of antibodies used for flow cytometry.

| **Marker** | **Fluorochrome** | **Host/Target** | **Isotype** | **Clone** | **Catalog Number** | **Dilution** |
| --- | --- | --- | --- | --- | --- | --- |
| **CD21** | APC/Cyanine7 | Mouse anti-human | IgG1, κ | Bu32 | 354928 | 1:400 |
| **Podoplanin** | Alexa Fluor® 647 | Mouse anti-human | IgG2a, κ | LpMab-21 | 395004 | 1:800 |
| **ICAM1 (CD54)** | Alexa Fluor® 700 | Mouse anti-human | IgG1, κ | HA58 | 353126 | 1:800 |
| **Thy1 (CD90)** | Brilliant Violet 650™ | Mouse anti-human | IgG1, κ | 5E10 | 328144 | 1:800 |
| **CD44** | Alexa Fluor® 594 | Mouse anti-human | IgG1, κ | CD44 | 397510 | 1:800 |
| **VCAM1 (CD106)** | PE/Cyanine 7 | Mouse anti-human | IgG1, κ | STA | 305818 | 1:400 |
| **CD157** | FITC | Rat anti-human | IgG2a, λ | W21007F | 382104 | 1:400 |
| **RANKL (CD254)** | PE | Mouse anti-human | IgG2b, κ | MIH24 | 347504 | 1:400 |
| **LTBR** | PE | Mouse anti-human | IgG2b, κ | 31G4D8 | 322008 | 1:400 |
| **CD35** | Brilliant Violet 421™ | Mouse anti-human | IgG1, κ | E11 | 333416 | 1:400 |
| **CD105** | PerCP/Cyanine5.5 | Mouse anti-human | IgG1, κ | 43A3 | 323216 | 1:400 |
| **CD4** | APC/Fire™ 750 | Mouse anti-human | IgG1, κ | SK3 | 980814 | 1:400 |
| **CD69** | Brilliant Violet 750™ | Mouse anti-human | IgG1, κ | FN50 | 310954 | 1:400 |
| **CD20** | PerCP | Mouse anti-human | IgG2b, κ | 2H7 | 302324 | 1:400 |
| **CD138** | Alexa Fluor® 700 | Mouse anti-human | IgG1, κ | MI15 | 356512 | 1:400 |
| **CD38** | Pacific Blue™ | Mouse anti-human | IgG1, κ | HB-7 | 356628 | 1:400 |
| **CD27** | APC | Mouse anti-human | IgG1, κ | O323 | 302810 | 1:400 |
| **Baff-Receptor** | PE/Dazzle™ 594 | Mouse anti-human | IgG1, κ | 11C1 | 316922 | 1:400 |
| **CD86** | Brilliant Violet 605™ | Mouse anti-human | IgG1, κ | BU63 | 374214 | 1:400 |
| **CD11c** | Brilliant Violet 785™ | Mouse anti-human | IgG1, κ | 3.9 | 301644 | 1:400 |
| **CD11b** | PE/Dazzle 594 | Mouse anti-human | IgG1, κ | ICRF44 | 301347 | 1:400 |
| **CD3** | FITC | Mouse anti-human | IgG2a, κ | OKT3 | 317306 | 1:800 |
| **CD68** | PE/Cyanine7 | Mouse anti-human | IgG2b, κ | Y1/82A | 333816 | 1:200 |
| **HLADR** | Brilliant Violet 421™ | Mouse anti-human | IgG2a, κ | L243 | 307636 | 1:200 |
| **CD30** | APC/Fire™ 750 | Mouse anti-human | Mouse IgG1, κ | BY88 | 333916 | 1:200 |
| **CD8** | Alexa Fluor® 700 | Mouse anti-human | IgG1, κ | SK1 | 344724 | 1:200 |
| **PD-1 (CD279)** | PerCP | Mouse anti-human | IgG1, κ | EH12.2H7 | 329938 | 1:200 |
| **CD19** | BV711 | Mouse anti-human | IgG1, κ | HIB19 | 302246 | 1:400 |
| **CD14** | PE | Mouse anti-human | IgG2a, κ | M5E2 | 301806 | 1:800 |


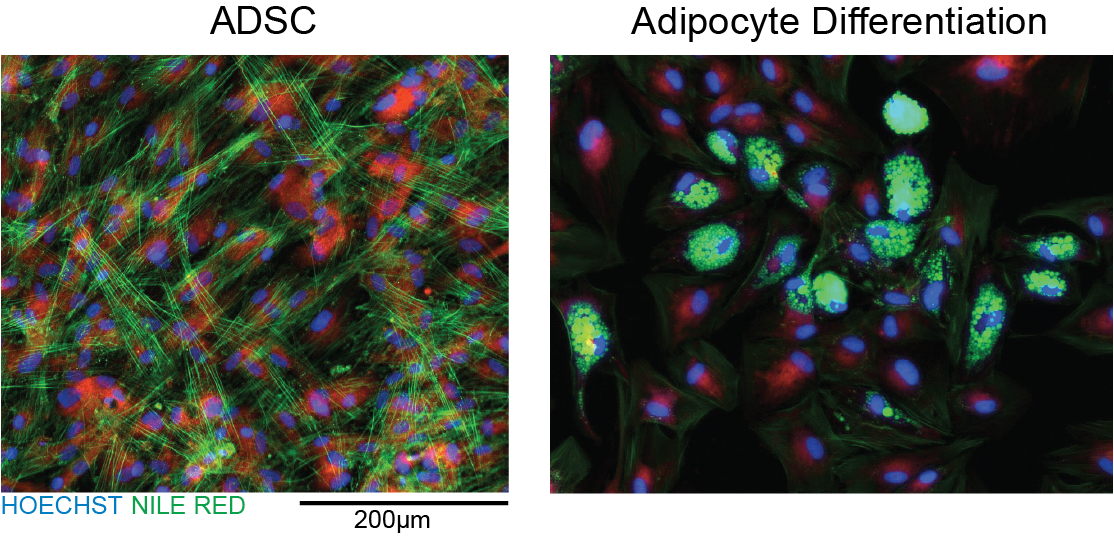


**Supplementary Figure 1:** Representative images of ADSCs and ADSCs differentiated into adipocytes after 10 days stained with Nile Red (green) and Hoechst (blue). Scale bar represents 200µm.


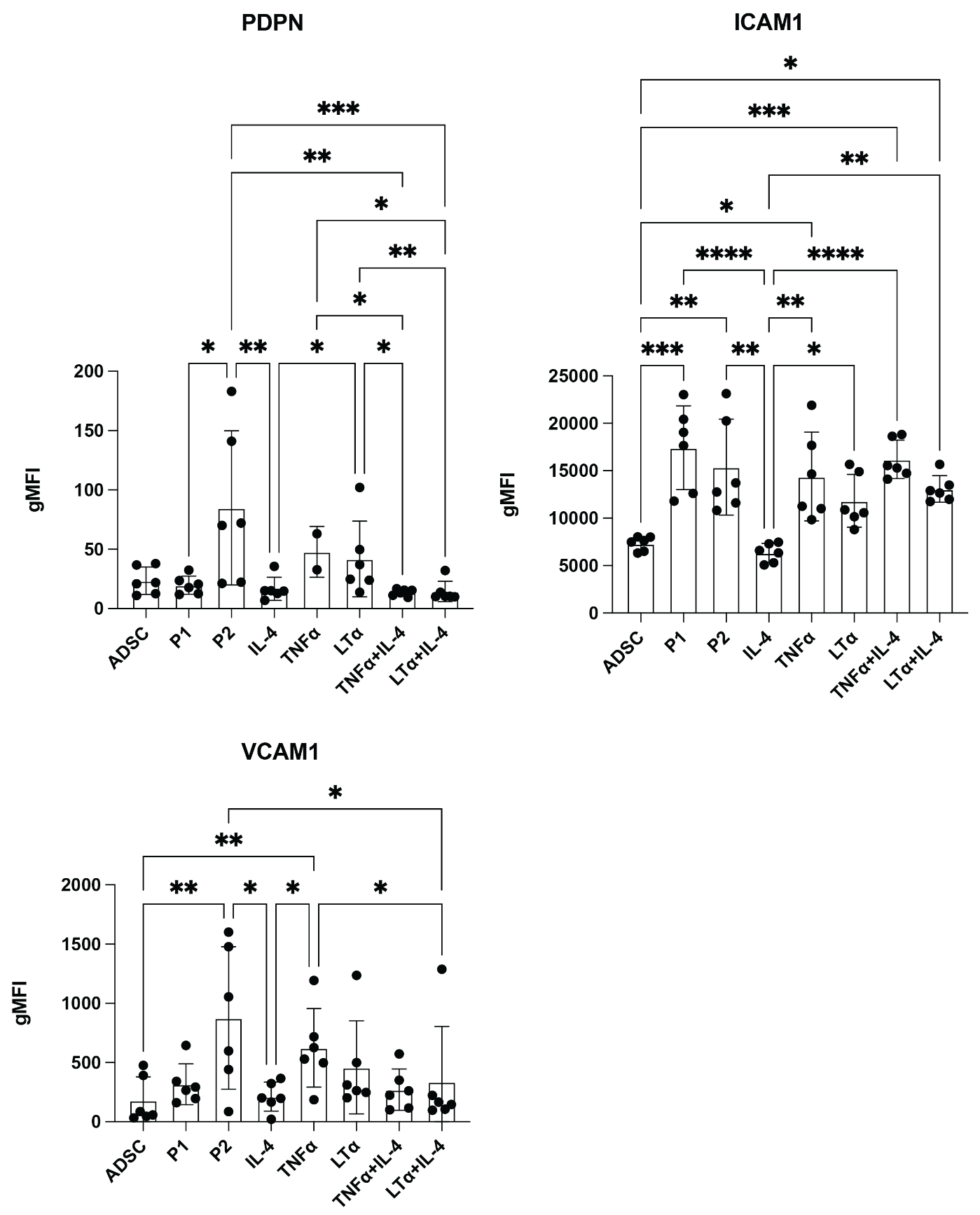


**Supplementary Figure 2:** Geometric mean fluorescence intensity (gMFI) of PDPN, ICAM1, and VCAM1 in total live populations of ADSC cells differentiated for 10 days using different cytokine combinations. Three unique ADSC donors were used. Data are shown as mean +/- SD. * indicates significant p ≤ 0.05. ** indicates significant p ≤ 0.01. *** indicates significant p ≤ 0.001. **** indicates significant p ≤ 0.0001.


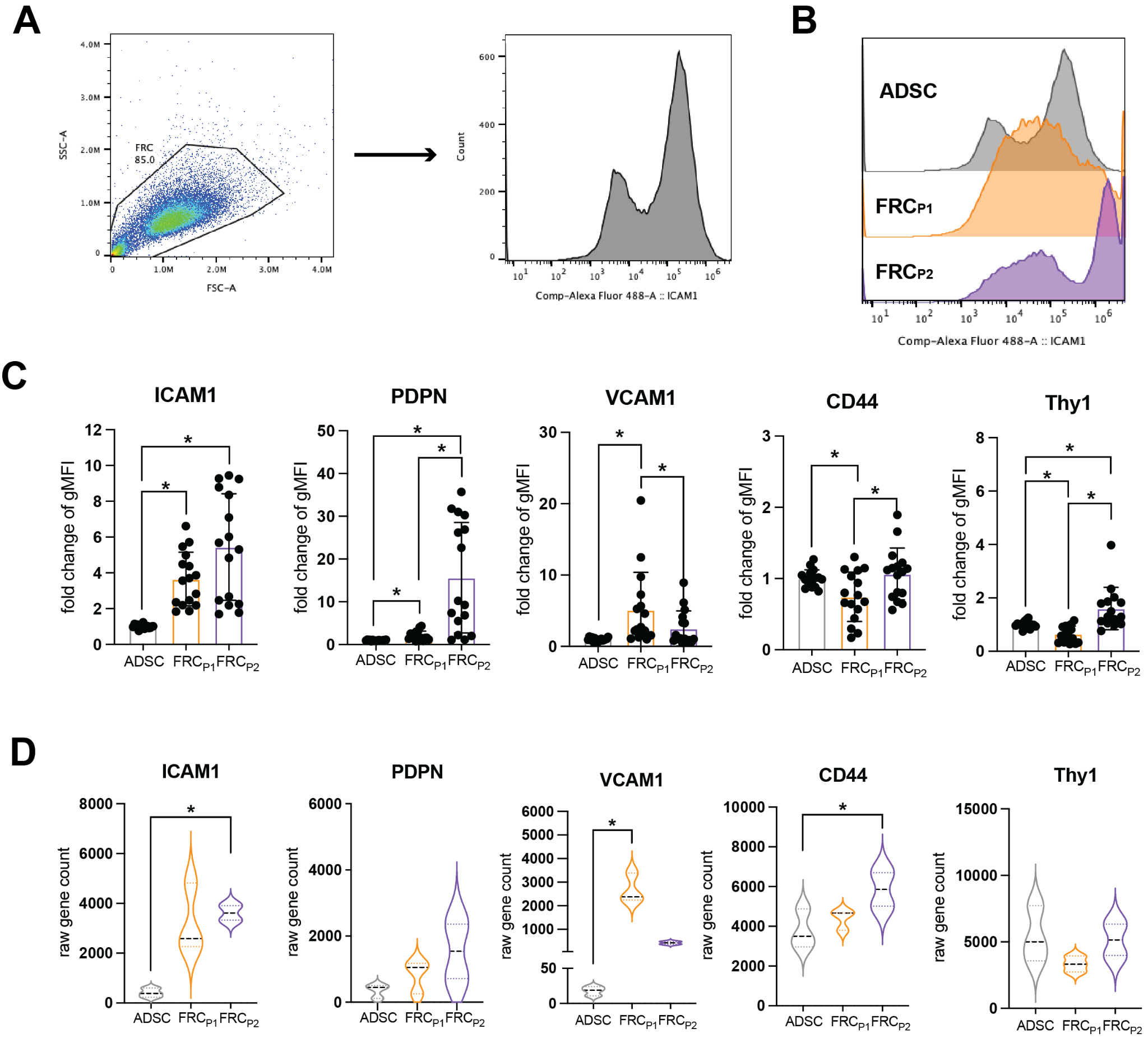


**Supplementary Figure 3:** **(A)** Example of gating strategy of FRC markers to get total geometric mean fluorescence intensity (gMFI) values from flow cytometry. **(B)** Representative comparison of expression of markers, using ICAM1, in the different cell populations. **(C)** Fold change of the gMFI of ICAM1, PDPN, VCAM1, CD44, and Thy1 in samples relative to ADSCs obtained using flow cytometry. **(D)** Raw gene count values of genes corresponding to flow cytometry data in ADSCs and FRC-like cells. Data are shown as mean +/- SD. * indicates significant p ≤ 0.05.


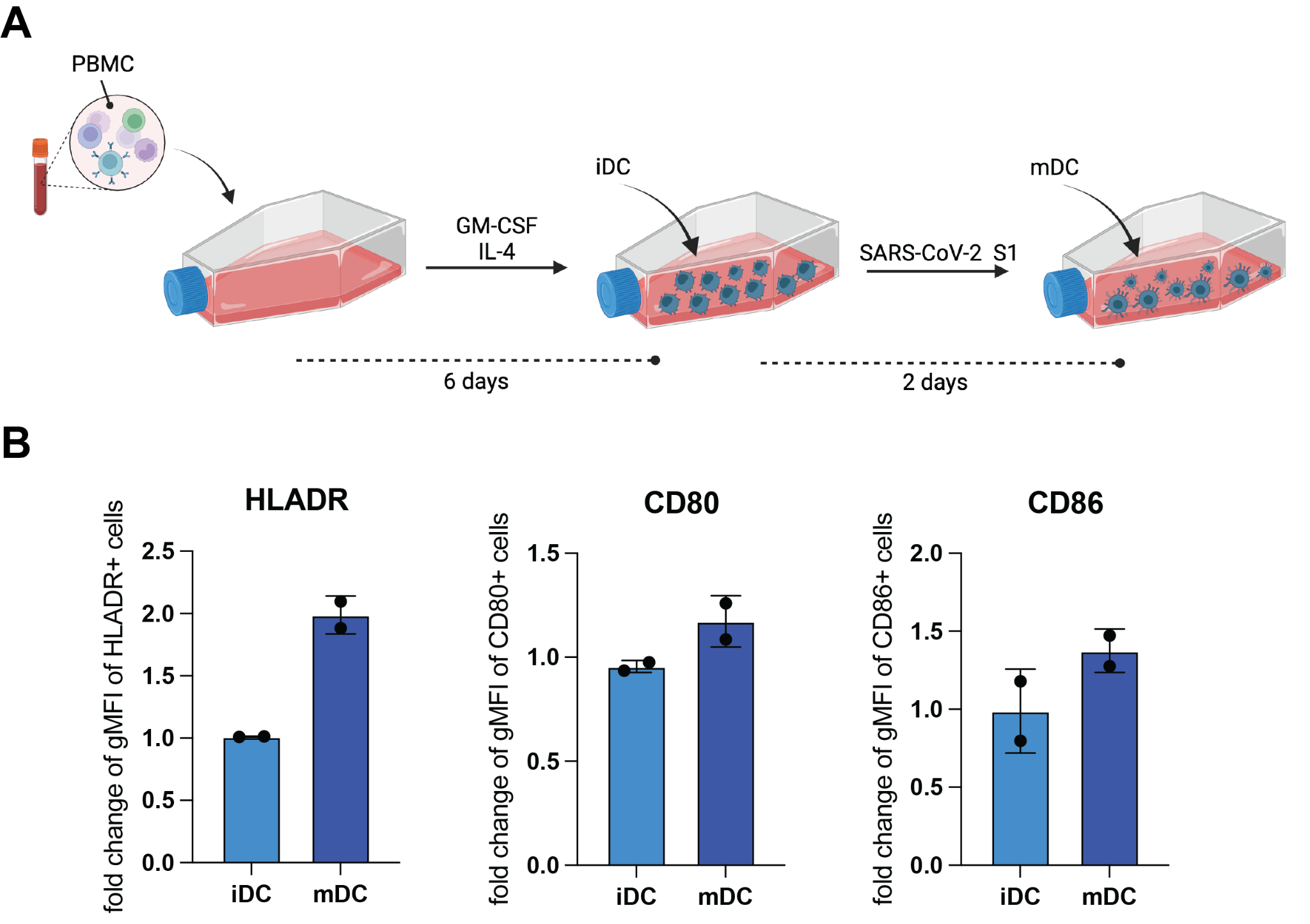


**Supplementary Figure 4:** (A) Differentiation of monocytes into iDCs by the addition of GM-CSF and IL-4 for 6 days. This is followed by the activation into mDCs using SARS-CoV-2 S1 spike protein for 2 days. (B) Flow cytometry results show an increase in the levels of HLADR, CD80, and CD86 in both PBMC donors used in the experiment. Data are shown as mean +/- SD.


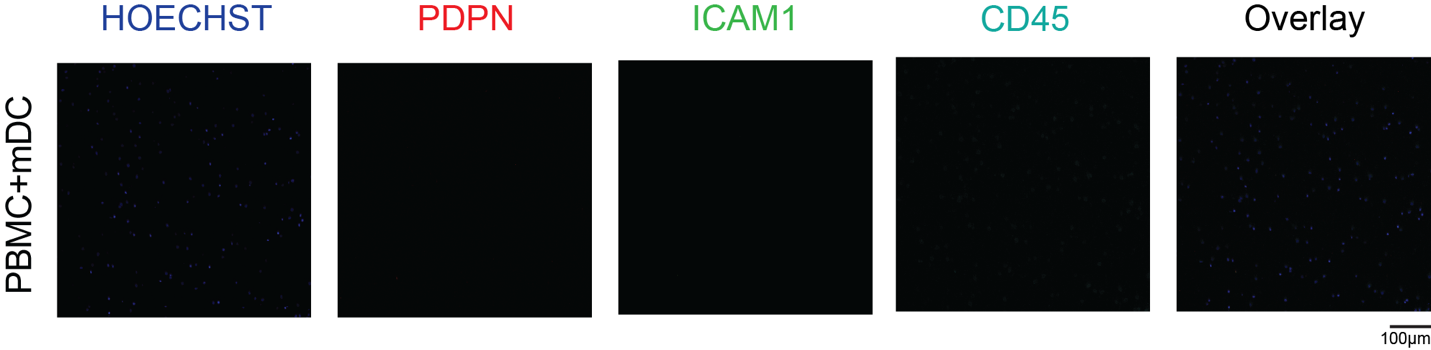


**Supplementary Figure 5:** Representative images of PBMC+mDC taken at 20x on Olympus. Samples were stained with Hoeschst (blue), PDPN (red), ICAM1 (green), and CD45 (cyan).


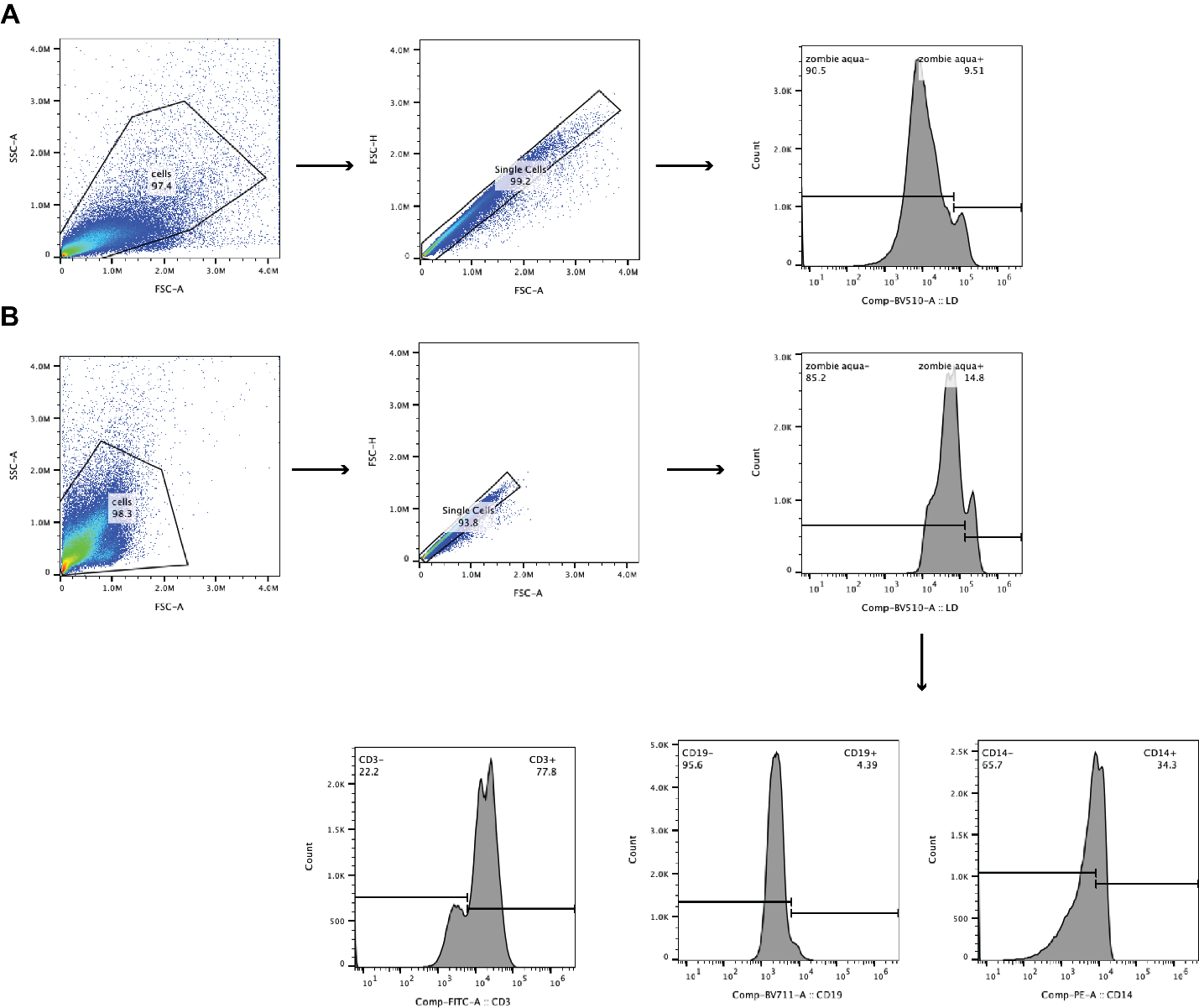


**Supplementary Figure 6:** **(A)** Representative gating of ADSC samples prior to UMAP analysis. Total cells gated, followed by single cell gating and selection of negative Zombie Aqua stain (live cells). **(B)** Representative gating of PBMC samples prior to UMAP analysis. Total cells gated, followed by single cell gating and selection of negative Zombie Aqua stain (live cells). Subsequently, each population (CD3+, CD19+, and CD14+) was gated and then processed.


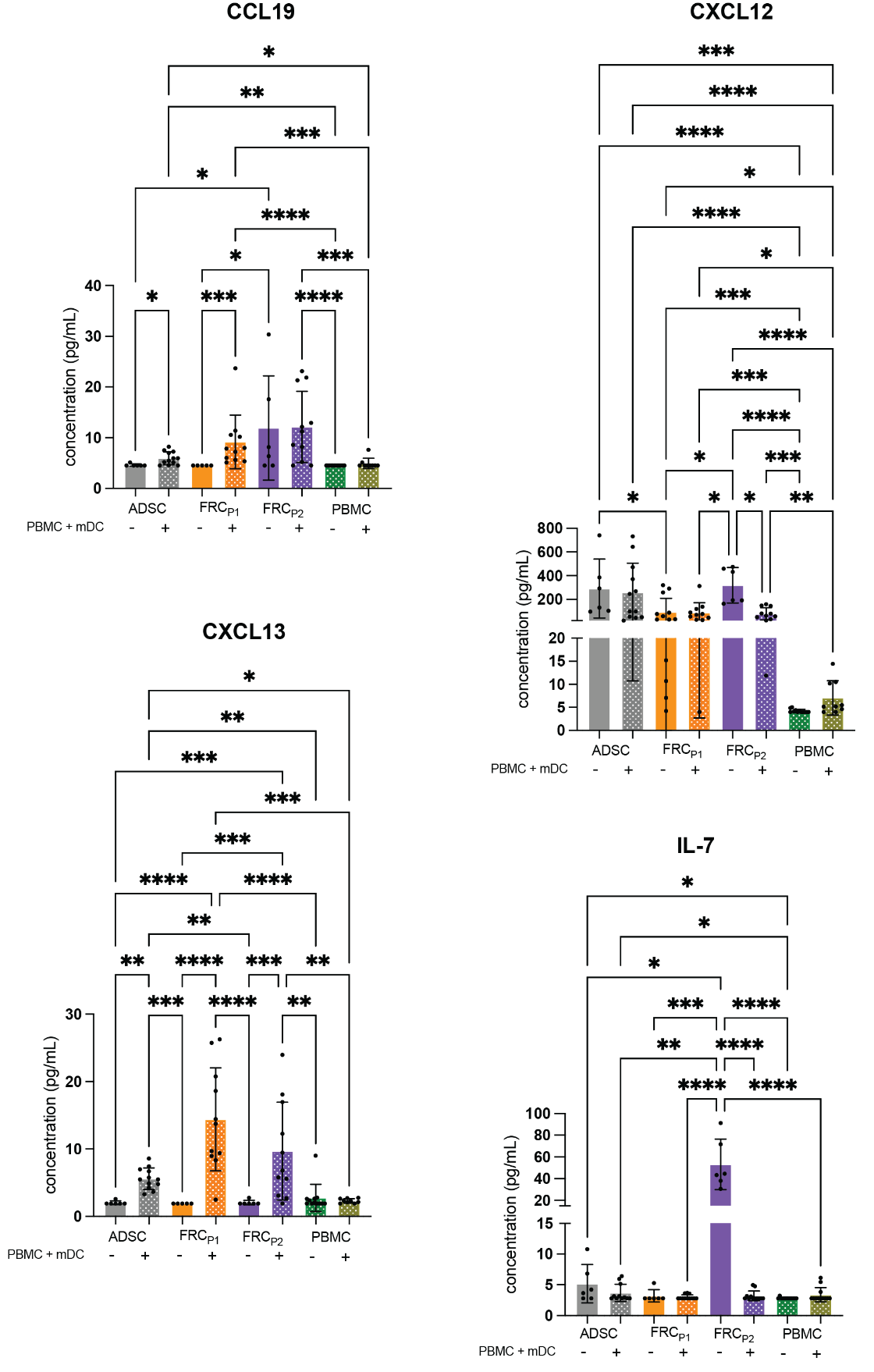


**Supplementary Figure 7:** Raw concentrations of chemokines and IL-7 produced by all conditions. 2 unique donors were used for PBMCs in all experiments and two unique ADSC donors were used. Data are shown as mean +/- SD. * indicates significant p ≤ 0.05. ** indicates significant p ≤ 0.01. *** indicates significant p ≤ 0.001. **** indicates significant p ≤ 0.0001.


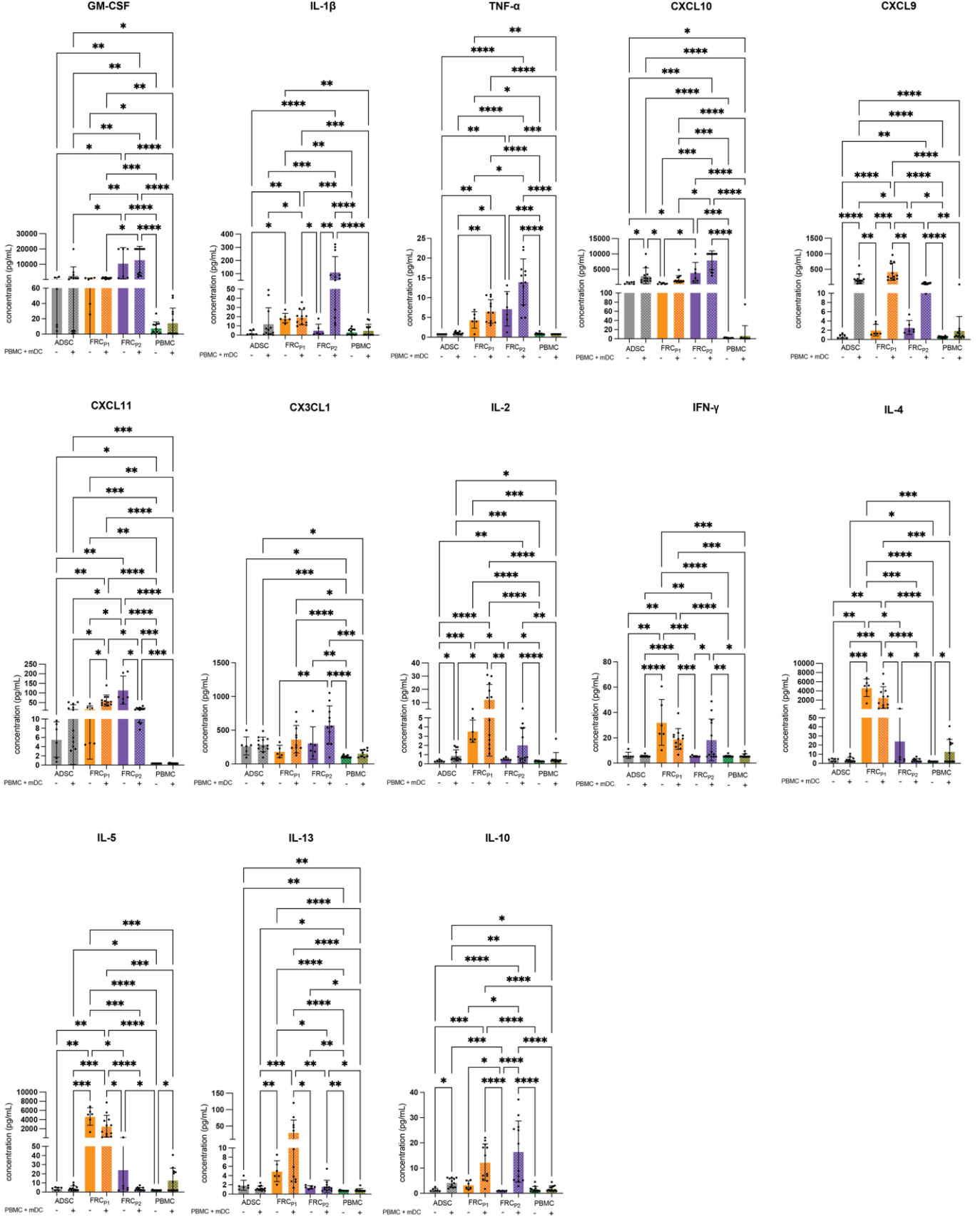


**Supplementary Figure 8:** Raw concentrations of chemokines and cytokines produced by all conditions. 2 unique donors were used for PBMCs in all experiments and two unique ADSC donors were used. Data are shown as mean +/- SD. * indicates significant p ≤ 0.05. ** indicates significant p ≤ 0.01. *** indicates significant p ≤ 0.001. **** indicates significant p ≤ 0.0001.

**
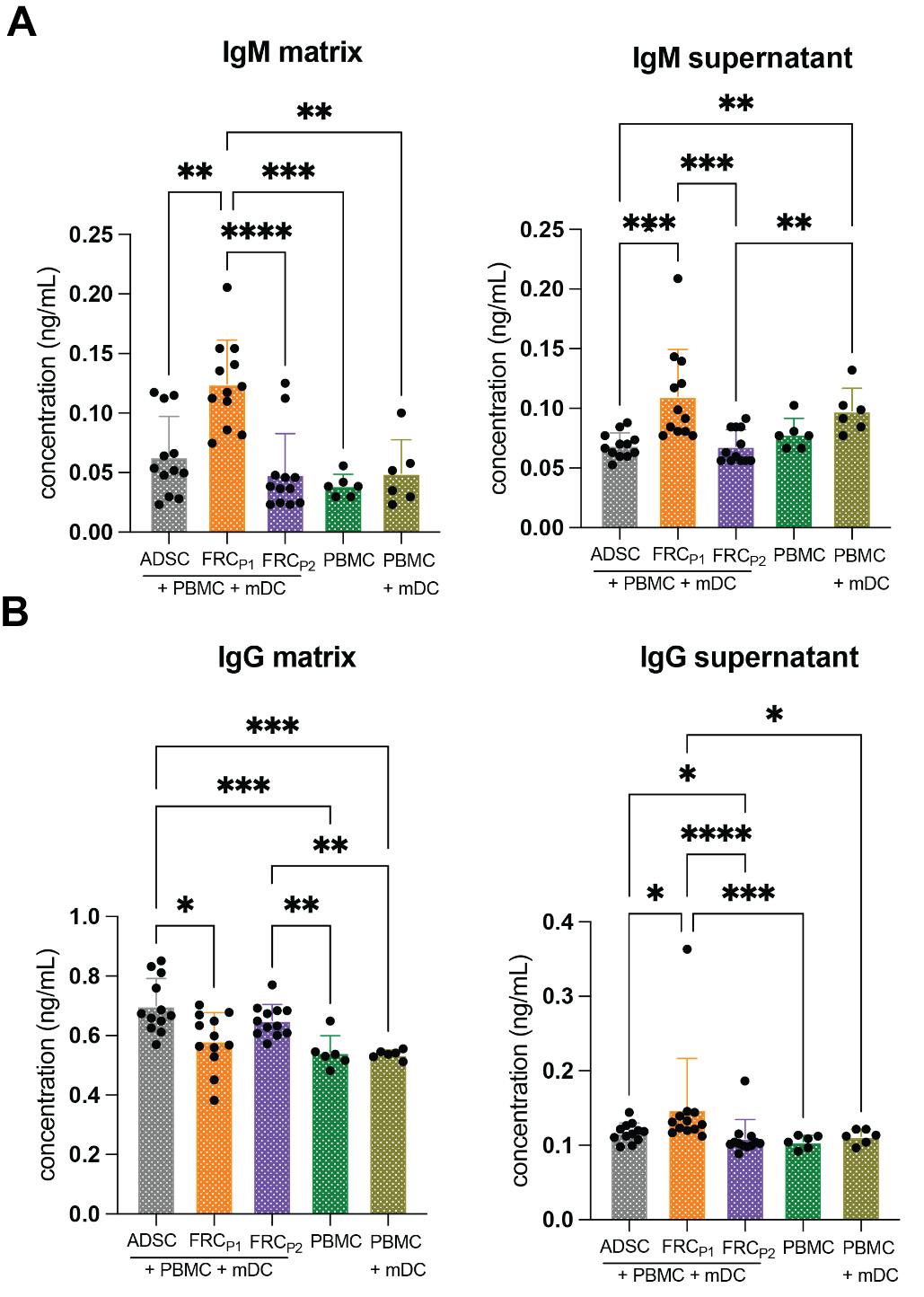
**

**Supplementary Figure 9:** Raw concentrations of IgM and IgG produced by co-culture conditions from the supernatant and matrices. 2 unique donors were used for PBMCs in all experiments and two unique ADSC donors were used. Data are shown as mean +/- SD. * indicates significant p ≤ 0.05. ** indicates significant p ≤ 0.01. *** indicates significant p ≤ 0.001. **** indicates significant p ≤ 0.0001.

<https://drive.google.com/drive/folders/1rvZfNhSAQZ9vOWMYBC3AME34TwCQaKku>

**Supplementary Video 1:** Z stack images of FRC_P1_ with PBMCs sample showing the dome-like structure. Images taken at 10X on LEICA STED stained with red (phalloidin) and blue (Hoeschst).
